# PABPN1-Dependent mRNA Processing Induces Muscle Wasting

**DOI:** 10.1371/journal.pgen.1006031

**Published:** 2016-05-06

**Authors:** Muhammad Riaz, Yotam Raz, Maaike van Putten, Guillem Paniagua-Soriano, Yvonne D. Krom, Bogdan I. Florea, Vered Raz

**Affiliations:** 1 Department of Human Genetics, Leiden University Medical Center, Leiden, The Netherlands; 2 Department of Molecular Epidemiology, Leiden University Medical Center, Leiden, The Netherlands; 3 Bio-organic Synthesis, Leiden Institute of Chemistry, Leiden, The Netherlands; 4 Department of Neurology, Leiden University Medical Center, Leiden, The Netherlands; The Jackson Laboratory, UNITED STATES

## Abstract

Poly(A) Binding Protein Nuclear 1 (PABPN1) is a multifunctional regulator of mRNA processing, and its expression levels specifically decline in aging muscles. An expansion mutation in PABPN1 is the genetic cause of oculopharyngeal muscle dystrophy (OPMD), a late onset and rare myopathy. Moreover, reduced PABPN1 expression correlates with symptom manifestation in OPMD. PABPN1 regulates alternative polyadenylation site (PAS) utilization. However, the impact of PAS utilization on cell and tissue function is poorly understood. We hypothesized that altered PABPN1 expression levels is an underlying cause of muscle wasting. To test this, we stably down-regulated PABPN1 in mouse *tibialis anterior* (*TA*) muscles by localized injection of adeno-associated viruses expressing shRNA to PABPN1 (shPab). We found that a mild reduction in PABPN1 levels causes muscle pathology including myofiber atrophy, thickening of extracellular matrix and myofiber-type transition. Moreover, reduced PABPN1 levels caused a consistent decline in distal PAS utilization in the 3’-UTR of a subset of OPMD-dysregulated genes. This alternative PAS utilization led to up-regulation of Atrogin-1, a key muscle atrophy regulator, but down regulation of proteasomal genes. Additionally reduced PABPN1 levels caused a reduction in proteasomal activity, and transition in MyHC isotope expression pattern in myofibers. We suggest that PABPN1-mediated alternative PAS utilization plays a central role in aging-associated muscle wasting.

## Author Summary

PABPN1 is a multifunctional regulator of mRNA processing and its levels are reduced in skeletal muscles from midlife onwards. Reduced PABPN1 levels in a mouse model causes muscle atrophy and muscle fiber switches. We show that PABPN1-regulated muscle atrophy is regulated, in part, by up regulation of Atrogin1 and reduced expression of proteasome genes via an alternative polyadenylation site utilization. This study reveals a functional role for alternative polyadenylation site utilization in muscle atrophy and suggests a role for RNA processing in muscle aging.

## Introduction

The landscape of mRNA in eukaryotic cells is partly maintained by mRNA processing at the 3’-UTR of transcripts [1]. mRNA processing is regulated by multiple RNA binding proteins, including poly(A) binding protein nuclear 1 (PABPN1), a regulator of poly-A tail length [2], mRNA decay [3] and proximal polyadenylation site (PAS) utilization at the 3’-UTR [4, 5]. An alanine expansion mutation in the N-terminus of PABPN1 is the genetic cause of OPMD, a late-onset myopathy [6]. Expanded PABPN1 forms intranuclear aggregates [7] entrapping additional nuclear proteins [8]. Whether these aggregates are toxic in physiological conditions is unsettled. Since insoluble aggregates reduce expression levels of soluble PABPN1 [9], it suggests that reduced levels of soluble PABPN1 could cause muscle weakness in OPMD. Despite the ubiquitous expression of PABPN1, in OPMD symptoms manifest predominantly in skeletal muscles from mid-life onwards [10]. PABPN1 expression levels in skeletal muscles are lower compared to other tissues [11]. As symptoms in OPMD predominantly affect skeletal muscles, others and we suggested that in muscles of OPMD patients PABPN1 levels decline below a critical threshold leading to molecular aberrations and cellular defects [12, 13]. Whether reduced PABPN1 levels leads to muscle pathology is not resolved yet.

Aging-associated muscle wasting is characterized by a decline in muscle strength and mass, which is accompanied by histological signatures of muscle weakness [14]. Aging muscles are also accompanied by transitions in myofibers, involving the switching between fast-twitch and slow-twitch myofibers [15]. Myofibers are classified by the expression of myosin heavy chain (MyHC) isotypes (MyHC-2b, -2x, -2a: fast-twitch; and MyHC-1) [16, 17]. This classification allows a detailed description of myofiber-type transition and could detect subtle changes in contractile properties of myofibers in response to external stimuli [18]. The patterns of MyHC isotype transition in response to mild changes in muscle environment are poorly understood. In addition, reduced muscle mass (atrophy) is also highly prominent in aging. Muscle atrophy is regulated by protein catabolism pathways, in particular the ubiquitin-proteasome system (UPS), which accounts for approximately 90% of protein turnover [19]. Up-regulation of ubiquitin ligases, in particular muscle-specific E3-ligases Atrogin-1/Fbxo32 (referred here as Atrogin-1) and Murf1, induces muscle atrophy by altering protein homeostasis [19–21]. Aging-associated reduction of proteasome activity results in accumulation of ubiquitinated protein and consequently and increase in protein aggregation [22].

Expression levels of the UPS genes are altered in late onset protein aggregation disorders [23]. We also found consistent dysregulation of the UPS gene network in models for OPMD [24–26]. Altered gene expression in a mouse model for OPMD is attributed to a switch from distal to proximal PAS utilization [4, 5] (referred here as alternative polyadenylation; APA). In cell cultures with PABPN1 knockdown, APA utilization was also reported [5]. We reported that PABPN1 expression levels decline from midlife onwards in skeletal muscles and reduced PABPN1 levels correlate with muscle symptoms in OPMD [27]. However, it is unclear whether mild reduction in PABPN1 levels, as found in OPMD and aging muscles, also causes APA utilization. Moreover, it is not resolved whether overexpression or reduced PABPN1 causes muscle pathology in OPMD. Others and we have suggested that reduced PABPN1 levels can cause muscle weakness [12, 13]. Here, we investigated the hypothesis that a mild reduction in PABPN1 levels impairs PABPN1 function and consequently induces muscle wasting. We examined primary molecular and histopathological alterations in TA with PABPN1 down regulation, and muscles. We show that a mild reduction in PABPN1 levels is sufficient to induce muscle wasting.

## Results

### Down regulation of Pabpn1 in mouse skeletal muscles

**Fig 1:**
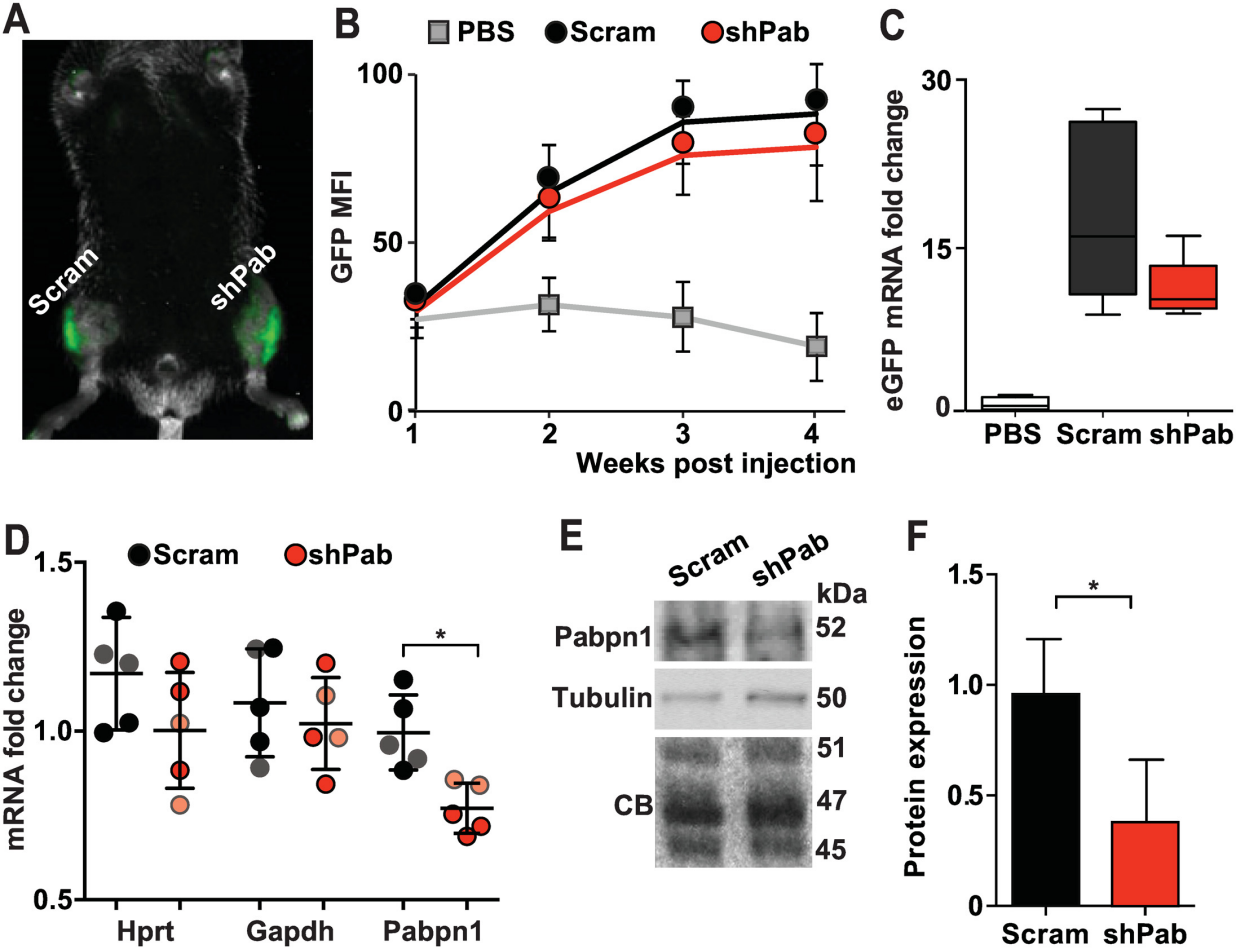
Pabpn1-DR in mouse TA muscles. **A.** A representative fluorescence image at four weeks post-injection of AAV containing shRNA construct to Pabpn1 or Scramble RNA. The GFP signal is localized into the injected TA muscles. **B.** GFP expression is stabilized three weeks post injection. **C.** Box plots show fold-change of eGFP mRNA expression in five mice and averages were normalized to PBS injected mice. **D-F.** Pabpn1 levels. **D.** Dot plot shows Pabpn1 mRNA Hprt and Gapdh levels in all injected mice. Hprt and Gapdh levels were normalized to mean Ct value in PBS injected mice. Pabpn1 levels were normalization to the mean of Gapdh and Hprt and PBS injected. Faded color circles mark mice with lower Pabpn1 levels. Means and SD are depicted with lines. **E.** A representative Western blot shows Pabpn1 and Tubulin proteins. Loading controls are denoted by Tubulin or Coomassie blue (CB) stained gel. **F.** Bar chart shows mean Pabpn1 accumulation is Scram and shPab muscles. Pabpn1 levels were normalized to the average of three proteins (45, 47 and 51 kDa) from the CB gel. Averages and SD are from three mice with lowest Pabpn1 levels. Statistical significance was assessed by paired Student’s t-tests. P-values <0.05 was considered statistically significant and indicated by an asterisk.

To mimic declining Pabpn1 expression levels during aging and OPMD, we stably down-regulated Pabpn1 by intramuscular injection of Adeno associated virus (AAV) serotype 9 expressing shRNA to Pabpn1 (shPab) into mouse TA muscles. As a control, AAV9 expressing scramble shRNA (Scram) was injected into contralateral TA muscles. Virus transduction was assessed by GFP fluorescence in living animals (Fig 1A). GFP fluorescence reached steady-state levels at three weeks post-injection (Fig 1B) and muscles were harvested at four weeks post-injection for *ex-vivo* analyses. Analyses of GFP fluorescence and eGFP mRNA expression levels showed comparable levels between shPab and Scram muscles (Fig 1C). This indicates that any differences between shPab and Scram muscles could be attributed to Pabpn1 levels and not to differences in transduction efficiencies. Analysis of Pabpn1 levels in shPab muscles revealed 20% reduction in mRNA levels (Fig 1D) and 50% reduction in protein levels (Fig 1E and 1F) as compared to Scram muscles. The expression levels of housekeeping genes, Hprt and Gapdh, remained unchanged (Fig 1D). A more prominent decrease in Pabpn1 protein levels compared to mRNA levels is in agreement with our previous observations of Pabpn1 down-regulation (DR) in muscle cell culture [25].

### Pabpn1-DR causes alternative polyadenylation in the UPS genes

**Fig 2:**
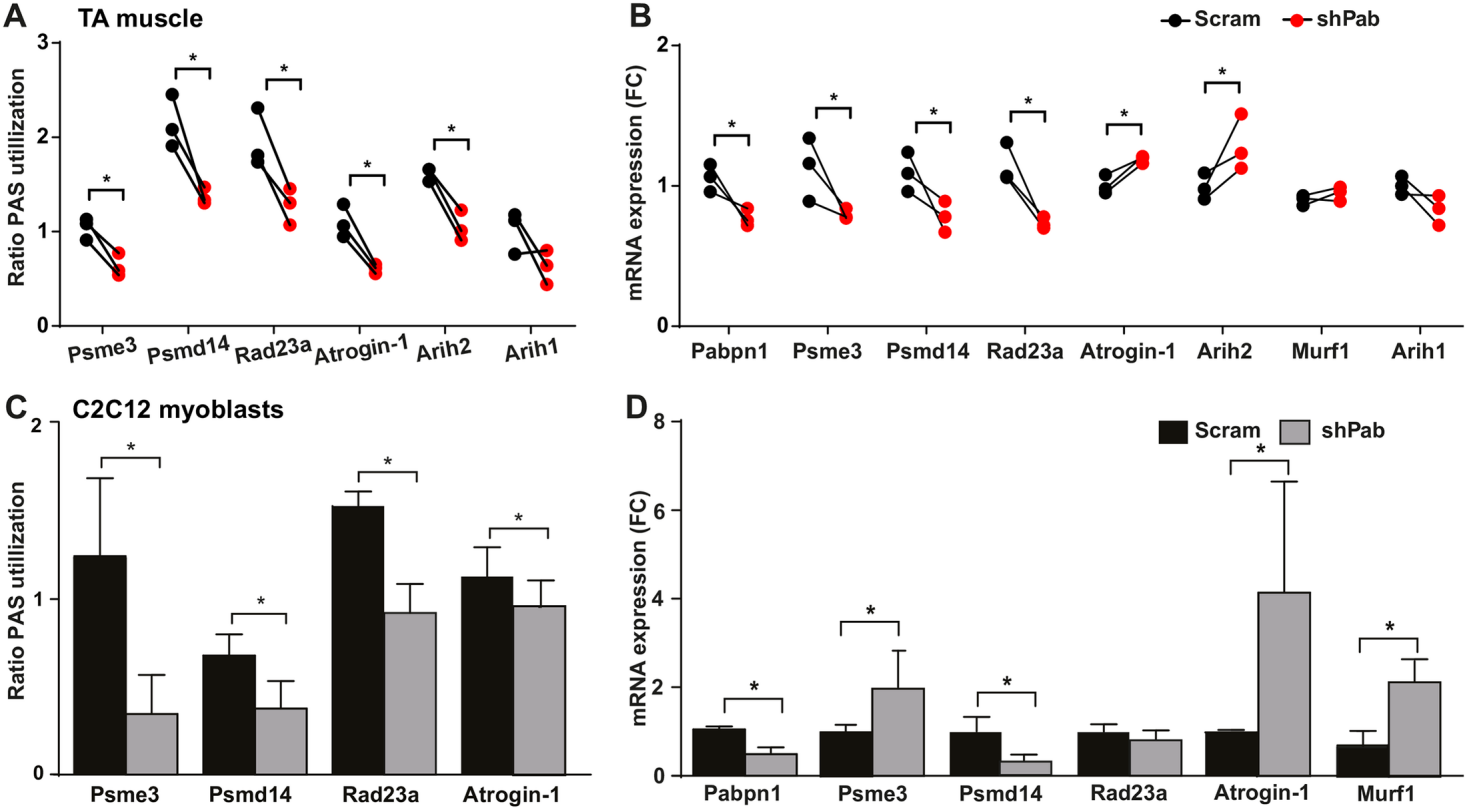
Pabpn1-DR causes a decrease in distal PAS utilization. **A.** Paired dot plot shows the change in PAS utilization between Scram and shPab muscles for 6 candidate genes; Arih2 PAS utilization is unchanged. **B.** Paired dot plot shows the mRNA fold change between Scram and shPab muscles. Murf1 and Arih1 levels are unchanged. **A-B.** Paired muscles are connected with a line. Statistical significance (P-value <0.05) was assessed by paired Student’s t-tests. **C-D.** PAS utilization and mRNA fold-changes in shPab cell culture. Mean and SD are from three biological replicates. Statistical significance (P-value <0.05) was assessed by unpaired Student’s t-tests. Fold changes were determined after normalization to *Hprt* housekeeping gene and to Scram muscles/cultures. PAS utilization was measured by ratio distal to total expression values. P-value <0.05 is depicted with an asterisk.

Pabpn1 regulates proximal PAS utilization at 3’-UTR of transcripts. We then investigated whether mild reduction in Pabpn1 levels causes a change in PAS utilization at the 3’-UTR. Eleven candidate genes whose mRNA levels are altered via PAS utilization in models for OPMD were selected for molecular investigation [4]. Protein catabolism machineries were previously found as most significantly dysregulated in OPMD [25], and therefore here we focus on genes of these networks including: E3-ligases: Atrogin-1, Murf1, Arih1, Arih2, Rbx1; proteasome associated genes: Psme3, Psmd14, Rad23a, Ubqulin1; and autophagy related genes: Atg10, Atg12. A change in PAS utilization and mRNA fold change was assessed with paired analyses between Scram and shPab muscles (Fig 2A and S1 Fig). A significant decline in distal PAS utilization in shPab muscles was found for six genes (Atrogin-1, Arih2, Psme3, Psmd14, Rad23a, Atg12). In five genes (Arih1, Rbx1, Cyld, Ubqln1 and Atg10), PAS utilization and fold change were unchanged in shPab muscles. A proximal PAS at the 3-UTR of Murf1 has not yet been identified. Importantly, in the three mice with lowest Pabpn1 levels a consistent reduction in distal PAS utilization was found (Fig 2A), affecting its expression levels (Fig 2B). In mice displaying milder Pabpn1-DR, a change in distal PAS utilization and mRNA expression were less pronounced (S1 Fig).

To validate that a moderate Pabpn1-DR affects PAS utilization and mRNA expression, the same shRNA was employed in C2C12 muscle cell cultures. Consistently, reduced distal PAS utilization was found for Psme3, Psmd14, Rad23a and Atrogin-1 (Fig 2C). Fold change direction in the myoblast culture was similar as in shPab muscles for Psmd14 and Atrogin-1 (Fig 2D). However Psme3 showed opposite fold change, Rad23a fold change was unchanged in cell culture, whilst Murf1 showed a twofold higher expression (Fig 2D). Together this indicates that Pabpn1 down regulation in muscles or in cell culture causes a consistent reduction in distal PAS utilization with varied fold change directions.

### Catalytic activity of β-subunits is impaired in Pabpn1-DR muscles

**Fig 3:**
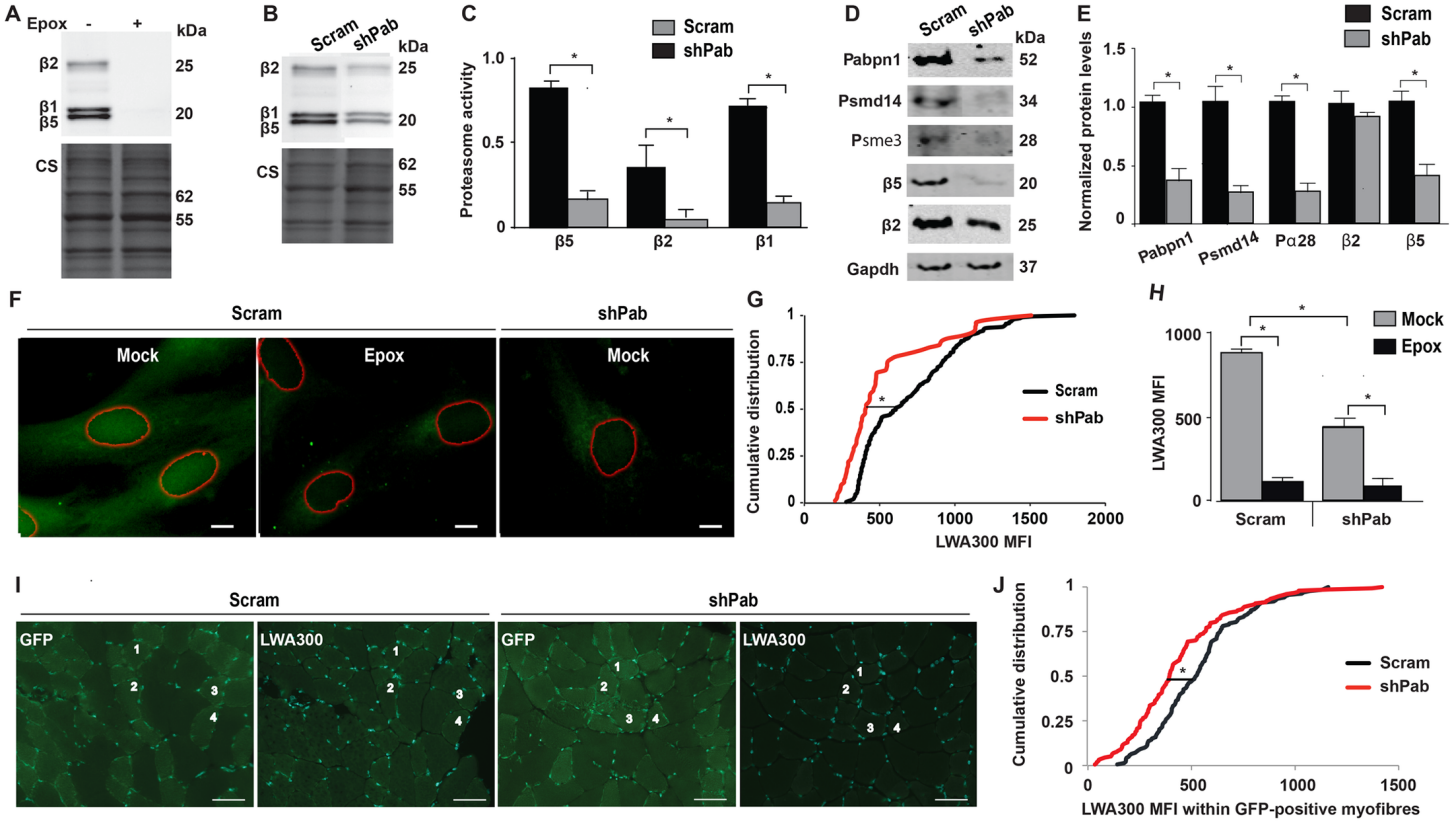
Proteasome activity in Pabpn1-DR cell culture and muscle tissue. **A-C.** Analyses of β-subunits catalytic activity in muscle cell culture using activity gel. Specific binding of LWA300 to β-subunits is demonstrated by pre-incubation with 0.5μM Epox for 1 hour (A). Proteasomal activity in Scram vs. shPab muscle cell cultures (B-C), activity gel shows a representative experiment (B) Equal loading was assessed by Coomassie staining (CS). Bar chart (C) shows LWA300 MFI in after normalization to CS. Averages are from triplicates. **D.** A representative western blot shows protein levels of proteasome-encoded genes in Scram and shPab cell cultures. **E.** Bar chart shows quantification of protein fold change after normalization to Scram cultures. Averages and standard variations are from three independent experiments. Statistical significance (p<0.05; *) was made with unpaired Student’s t-test. **F.** Images show subcellular localization of LWA300 in Scram or shPab cell cultures. Cultures were incubated with 62.5 nM LWA300, pre-incubation with 0.5μM Epox was carried out as control. Segmented nuclei are depicted with red circles. Scale bar is 5 μm. **G.** Plot shows cumulative distribution of nuclear LWA300 MFI in Scram or shPab C2C12 cells. Statistical significance (p<0.05) was assessed with the Kruskal-Wallis test. **H.** Bar chart shows flow cytometer analysis of LWA300 MFI in Scram or shPab cell cultures treated with or without Epox. Averages are from three experiments. Representative histogram of flow cytometer output is in S2 Fig. **I-J.** LWA300 signal in muscle cryosections. **I.** Images show GFP or LWA300 fluorescence in successive cryosections from Scram or shPab. Examples of the four same myofibers in GFP and LWA300 are depicted with numbers. Nuclei were counterstained with DAPI. Scale bar is 50 μm. **J.** Plot shows cumulative distribution of nuclear LWA300 MFI in cryosections from three mice. Over 400 myofibers are included per condition per mouse. Statistical significance (p<0.05) was assessed with Kruskal-Wallis test.

In a previous study we reported that in OPMD proteasomal gene network is highly dysregulated [26]. To investigate whether Pabpn1-mediated changes of the proteasome gene network affect proteasome activity we measured proteasomal activity using the pan-reactive activity-binding probe, LWA300. LWA300 specificity in muscle cell culture was demonstrated by pre-incubation with Epoxomicin (Epox) a binding competitor. Reduced LWA300 signal was found for β5, β1 and β2 subunits (Fig 3A), indicating specific binding to these proteasomal subunits. Also in shPab myoblast culture LWA300 signal was decreased compared with control culture (Fig 3B and 3C). The reduced activity of β5-subunit is caused, in part, by reduced β5 protein expression (Fig 3D and 3E). However, reduced activity of β2 is not corroborated with reduced protein levels (Fig 3E). In addition, levels of Psmd14, and Psme3 were also reduced in shPab cell culture.

We next assessed whether Pabpn1 levels affect LWA300 subcellular distribution. In control myoblasts LWA300 signal was localized into both the cytoplasm and the nucleus (Fig 3F). Epox pre-treatment reduced LWA300 signal in both nuclear and cytoplasmic compartments, though some unspecific staining was observed in the cytosol (Fig 3F). This unspecific binding is consistent with the lipophilic properties of the LWA300 probe, and binding to the endoplasmic reticulum [28]. Notably, in the shPab myoblasts LWA300 signal was dramatically reduced in the nucleus (Fig 3F and 3G). Since unspecific binding of LWA300 was minimal in living cells, we then assessed whether changes in LWA300 mean fluorescence intensity (MFI) could be measured using the flow cytometer. Epox pre-treatment reduced LWA300 MFI, and consistently reduced LWA300 signal was measured in shPab cultures compared with control (Fig 3H and S2 Fig). Together, this reveals that Pabpn1 levels affect proteasome activity in muscle cells.

We then investigated whether proteasome activity can be also measured in shPab muscles. We optimized a protocol to detect LWA300 specific signal in muscle cryosections (S3 Fig) and subsequently, the effect of reduced Pabpn1 levels on proteasome activity was measured only in the GFP positive myofibers (Fig 3I). Images showed that GFP positive myofibers had lower LWA300 signal compared with GFP negative myofibers Fig 3I). Indeed, cumulative distribution of LWA300 MFI revealed reduced signal in shPab compared with Scram transduced myofibers (Fig 3J). Together this indicates that reduced Pabpn1 levels in skeletal muscles impair proteasome activity.

### Pabpn1-DR induces muscle pathology

**Fig 4:**
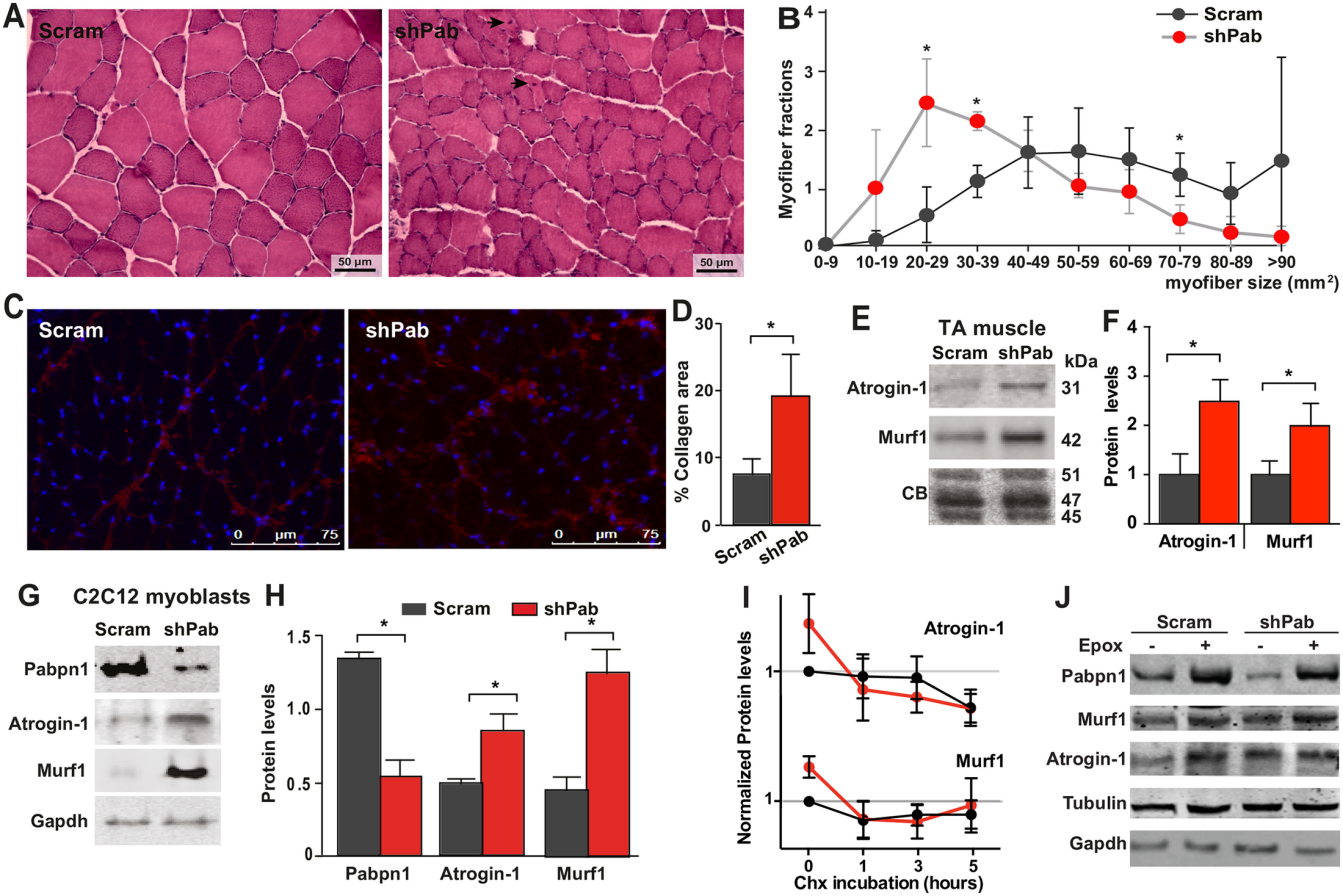
Histopathological characteristics of Pabpn1-DR muscles. **A-B.** Haematoxylin and eosin staining of muscle tissue. Myofibers with central nucleation are indicated with black arrows. Scale bar is 50 μm. B. Quantification of myofiber cross-sectional area. **C-D.** Quantitation of thickening of ECM in Pabpn1-DR muscles. (C) Shown are representative images of muscle sections stained for Collagen 1 and counterstained with DAPI. Scale bar is 75 μm. (D) Percentage of collagen positive area of muscle sections. Bars show SD from three mice. **E-F.** Western blot (E) shows Atrogin-1 and Murf1 protein levels in Scram and shPab muscles. Bar charts (F) show means of Atrogin-1 and Murf1 levels normalized to the average of three proteins (45, 47 and 51 kDa). Averages and SD are from three mice. **G-H.** Atrogin1 and Murf1 protein levels in Scram and shPab cell cultures. Gapdh is used as loading control. Averages and SD are from three biological replicates. Statistical significance was assessed by paired student’s t-test. P-value <0.05 was considered statistically significant and indicated by an asterisk. **I.** Dot plots show protein accumulation of Atrogin-1, Murf1 in Scram (in black) or shPab (in red) cell cultures after Chx treatment for 0, 1, 3, 5 hours. Protein levels were normalized to untreated in Scram cultures (time 0). Averages and standard deviations are from three independent experiments. Equal loading was assessed by Tubulin and Gapdh (representative blots are shown in S5 Fig). **J.** Western blot shows protein accumulation in mock or Epox treated myoblasts cultures. Blots were incubated with antibodies to Pabpn1, Murf1, Atrogin-1, or Gapdh and Tubulin as loading controls.

We next investigated whether reduced Pabpn1 levels also affect muscle pathology at 4 weeks post-injection. Haematoxylin and eosin staining revealed that shPab muscles were not severely degenerated and only few myofibers were exhibiting central nucleation (Fig 4A). However, we found reduced cross sectional area (CSA) of myofibers in shPab muscles compared with Scram muscles (Fig 4B). In addition, we quantified thickening of the extracellular matrix (ECM) in shPab muscles (Fig 4C and 4D). In contrast, fatty infiltration and up-regulation of inflammatory genes were not found in shPab muscles (S4 Fig).

Muscle atrophy is characterized by reduced myofiber CSA, central nucleation and thickening of the ECM, and is regulated by Atrogin-1 and Murf1 [19]. Western blot analyses revealed significant increase in Atrogin-1 and Murf1 levels in shPab muscles (Fig 4E and 4F). An increase in both proteins was also found in shPab myoblast culture (Fig 4G and 4H).

Since proteasome activity in shPab muscles is reduced, we therefore expected an aberrant protein accumulation in shPab muscles compared to scram muscles. To investigate this we treated shPab and scram cell cultures with Cycloheximide (Chx), which inhibits the ribosomal machinery. We found differential patterns of Atrogin-1 or Murf1 protein accumulation between shPab and scram cell cultures (S5 Fig). Reduced Atrogin-1 levels were found after 3 hours Chx treatment whilst in shPab culture Atrogin-1 levels declined after only 1 hour (Fig 4I). In contrast, the pattern of Murf1 accumulation in response to Chx treatment was alike in both Scram and shPab cultures (Fig 4I). Levels of Gapdh, loading control, remained unchanged (Fig 4I). This indicates that Atrogin-1 protein turnover is affected by limited Pabpn1 levels, whereas Murf1 is less affected. Moreover, inhibition of the proteasome using Epox treatment resulted in differences in protein accumulation between control and shPab cultures (Fig 4J). Epox treatment in control cells caused increased accumulation of Pabpn1, Atrogin-1 and Murf1 proteins. In shPab cultures, levels of Atrogin-1 and Pabpn1 showed higher accumulation compared with controls, whereas Murf1 levels were unchanged (Fig 4J). This suggests that levels of Atrogin-1 and Pabpn1, but not Murf1, are up regulated in part by the proteasome in shPab conditions.

### Pabpn1-DR leads to myofiber-type transition

**Fig 5:**
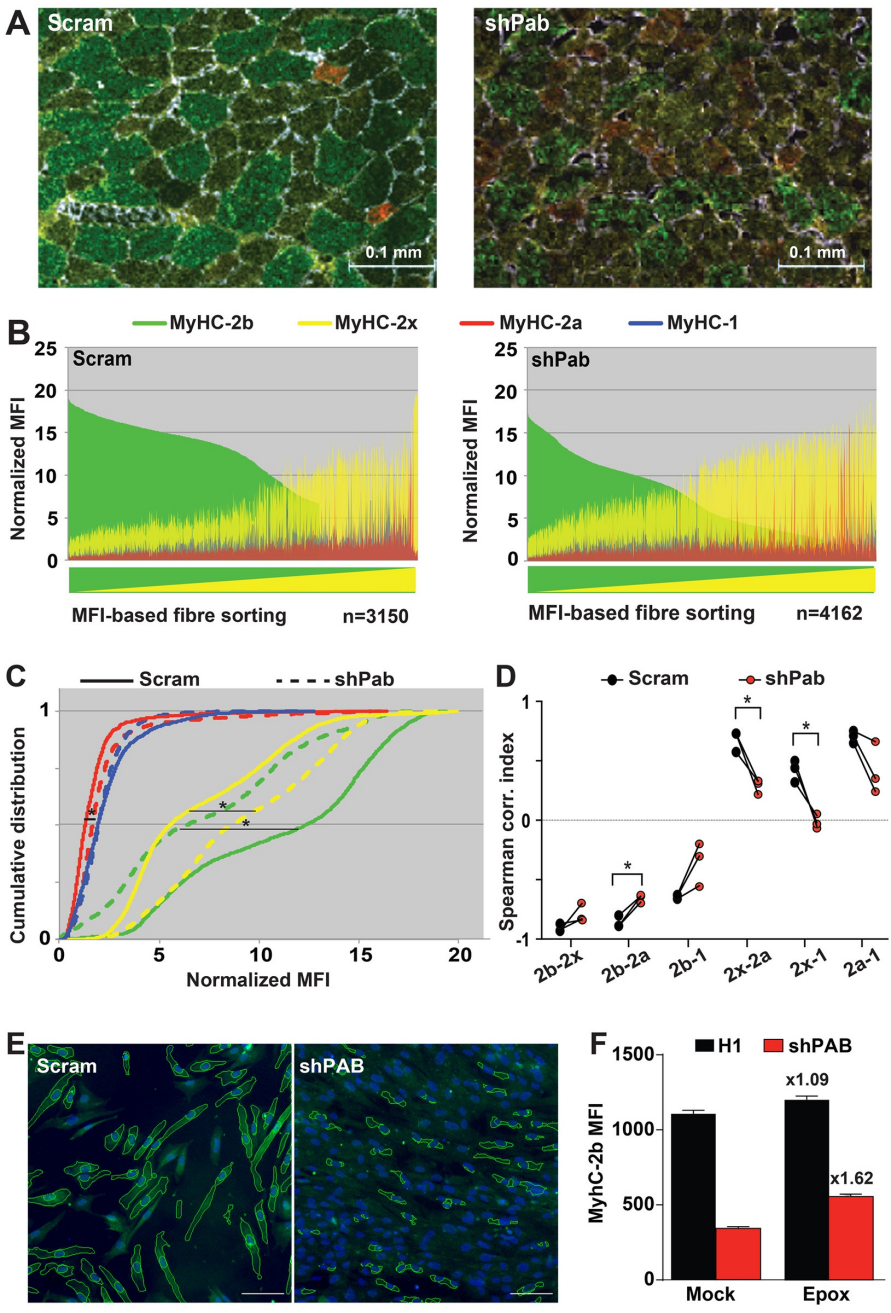
Papbn1-DR causes muscle fiber-type transition. **A.** Merged images of muscle cross sections stained with antibodies to four MyHC (type -2b in green; -2x in yellow; -2a in red; and type-1 in blue) isotypes and to laminin. Scale bar is 100 μm. **B.** MFI distribution plots show the distribution of each MyHC isotype MFI per myofiber in Scram or shPab muscles. Distribution plots were made from N >3000 myofibers. Myofibers were sorted by MyHC-2b (high to low) and MyHC-2x (low to high). **C.** Plots show cumulative distribution of MFIs of MyHC isotypes per myofiber between Scram and shPab muscles. **D.** Changes in Spearman correlation indices between two MyHC isotypes in shPab muscles as compared with the Scram muscles. Statistical significance was assessed by a paired Student’s t-test from N = 3 mice and paired muscles per mouse are connected with a line. The differences in MFI distribution between scram and shPab muscle were statistically evaluated using Kruskal-Wallis test. P-value <0.05 was considered statistically significant and indicated by an asterisk. **E-F.** Epox treatment affects MyHC expression in shPAB cultures. 7304.1 human cultures were fused for three days and subsequently were incubated with Epox. MyHC-2b expression was determined with immunofluorescence, and stained cultures were imaged and MFI was quantified in segmented cytoplasmic regions (E). Bar chart (**F**) shows MFI quantification, means and standard errors of the means are from 3760 ± 1870 myotubes. The fold change between mock and Epox treatments is indicated above the bars.

To assess whether reduced Pabpn1 levels also affect myofiber contractile properties a quantitative immunohistology procedure of four MyHC isotypes was applied and MFI was statistically analysed (Fig 5A and S6 Fig). Paired analyses were carried out in the three mice with lowest Pabpn1 levels. Distribution plots of MyHC MFI in myofibers suggested a decrease in MyHC-2b but an increase in MyHC-2x in all three mice (Fig 5B and S7 Fig). A change in MyHC MFI between shPab and Scram muscles was then assessed with cumulative distributions revealing a significant decrease in MyHC-2b and an increase in MyHC-2x in shPab muscles (Fig 5C). Quantitative analysis of MyHC-2a MFI revealed a small but significant increase in shPab muscles compared with Scram muscles (Fig 5C). MyHC-1 levels remained unchanged (Fig 5C). Moreover, with this procedure we noted that in most myofibers at least two MyHC isotypes co-expressed (Fig 5B). We then assessed whether Pabpn1 levels affect MyHC co-expression. In control muscles we found strong negative correlations between MyHC-2b and all other MyHC isotypes. However, in shPab muscles correlations were significantly weaker (Fig 5D and S1 Table). In contrast, positive correlations between MyHC-2x and MyHC-2a or MyHC-1 were found in control muscles, but a weaker correlation was found in shPab muscles (Fig 5D and S1 Table). This suggests that reduced MyHC-2b expression in shPab muscles is associated with an increase in MyHC-2x and/or MyHC-2a co-expression. Together these data suggest that reduced Pabpn1 levels induce transitions in myofiber-types from fast to slower-twitch myofibers. In contrast to the effect on protein levels of MyHC isoforms, reduced Pabpn1 levels did not affect mRNA expression of MyHC genes (S8 Fig).

We then examined whether Pabpn1-mediated changes in MyHC isotype expression could be affected by the impaired proteasome activity in shPab muscles. We tested MyHC expression in human 7304.1 muscle culture. In agreement with the findings in shPab muscles, MyHC-2b was also reduced in shPAB cultures (Fig 5E and 5F). Epox treatment caused only a small increase (1.09 fold) in MyHC-2b MFI in control culture, but in shPab culture this increase in MyHC-2b MFI was 50.1% higher than in control culture, representing 1.63 fold over mock (Fig 5F). The expression of MyHC isotypes type-2x, -2a and type-1 in these human myotube cultures was under detection levels. These data suggest that Pabpn1-associated myofiber transitions could in part be mediated through proteasome activity.

## Discussion

PABPN1 levels are reduced in muscles during normal aging and also in OPMD disease [27]. Whether a change in PAS utilization by PABPN1 levels has an impact on cell and tissue function is poorly studied. Moreover the effect of reduced PABPN1 levels in skeletal muscles has not been investigated. Here, we generated a mouse model to investigate a role for reduced Pabpn1 levels in aging associated muscle pathology. We show that mild (20–50%) down regulation of Pabpn1 is sufficient to cause APA utilization. We focused on a model with only mild PABPN1-DR, aiming to study the initial effect of reduced PABPN1 levels in muscles. Genome-wide APA utilization at the 3’-UTR of the transcripts was initially found in the A17.1 OPMD mouse model [4, 5]. This mouse model was generated by high overexpression (30-fold) of expanded PABPN1 [29], and was instrumental demonstrating causality to muscle pathology. However, overexpression of PABPN1 was not reported in heterozygous OPMD patients. There are two prevailing disease mechanisms in OPMD, as for many protein aggregation disorders, toxic gain of function versus loss of function. A toxic gain of function is concluded from overexpression models. More recently, evidences, including ours, support that loss of PABPN1 function, either due to reduced expression level [27] or depletion of the soluble protein [9] is a plausible disease model for OPMD. Our results here further demonstrate, using quantitative assays, that reduced PABPN1 levels PABPN1 cause muscle pathology, including atrophy, and ECM thickening. In a recent study we showed the ECM thickening is found in OPMD muscles and PABPN1 overexpressing models for OPMD [30]. To assess that PABPN1 function is affected in shPab muscles, we determined PAS utilization in 11 genes whose expression levels are dysregulated in models overexpressing expPABPN1. From these, reduced distal PAS utilization and gene dysregulation were found for only 6 genes. Reduced distal PAS utilization is consistent between shPab and A17.1 muscles. In addition, Murf1 levels were dysregulated in A17.1 muscles but not in shPab muscles [24]. These differences could be attributed to differences between the two models: in A17.1 expPABPN1 is constitutively expressed under a strong promoter, and muscles were examined after 6, 18 and 26 weeks [24] compared with our shPab model. It is also possible that functional Pabpn1 levels in the A17.1 mouse are below that in shPab muscles.

**Fig 6:**
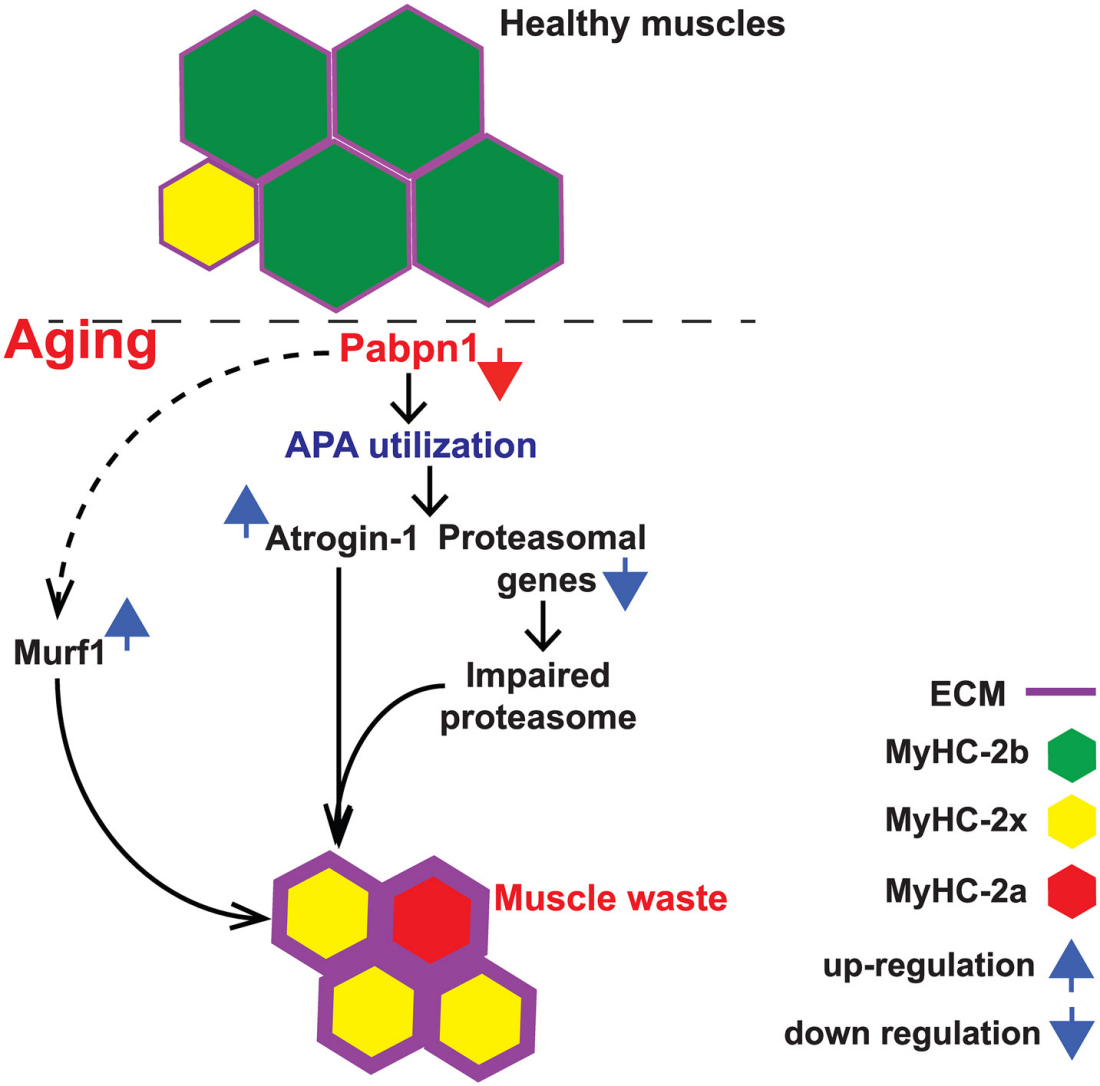
A model for the effect of reduced PABPN1 levels on muscle atrophy. Reduced PABPN1 levels induced a alternative polyadenylation site (APA) utilization in the 3’-UTR of a subset of transcripts whose gene product regulates muscle waste, including Atrogin-1/Fbxo32 and proteasomal genes. In these muscles, Atrogin-1 is overexpressed, whilst proteasome activity is reduced. This leads to pathological hallmarks of muscle atrophy including reduction in myofiber cross-sectional area, increase in extracellular matrix (ECM) and a switch in myosin heavy chain (MyHC) expression pattern in myofibers.

Atrogin-1 and Murf1 are key regulators of muscle atrophy [19, 21]. In shPab muscles we found muscle atrophy together with up-regulation of Atrogin-1 and Murf1 proteins. We show that an increase in Atrogin-1 protein levels in shPab conditions is in part caused by a change in PAS utilization and faster protein turnover. This suggests that Atrogin-1 transcript levels are directly regulated by PABPN1 (Fig 6). In addition, Atrogin-1 protein stability is affected by genes of protein catabolism network whose levels are regulated by PABPN1. Specifically, we found that reduced proteasome activity in shPab conditions affect Atrogin-1 levels but not Murf1. Reduced Pabpn1 levels have a lesser effect on Murf1 dysregulation. In the A17.1 mouse model we found an increase in Murf1 mRNA [24]. Here we show that Murf1 mRNA levels were unchanged by PABPN1 levels in muscles, whereas in cell culture Murf1 mRNA were up regulated. This could suggest that Murf1 mRNA levels could be affected by a greater loss of Pabpn1. Alternatively, it is also possible that unlike Atrogin-1, which seems to be directly regulated by PABPN1 levels, PABPN1 levels indirectly regulate Murf1 levels.

In contrast to transcript up-regulation via PAS utilization in Atrogin-1, for the proteasome genes reduced distal PAS utilization led to reduced expression levels in muscles. Consistently, reduced PABPN1 levels caused reduced distal PAS utilization in both muscles and cell cultures, however fold change direction was not always consistent: Psme3 transcript was down-regulated in muscles, and up-regulated in cell culture, Rad23b was down-regulated in muscles but its levels were unchanged in cell culture. This suggests that mRNA fold changes are not solely determined by PAS utilization. Up-regulation of transcripts with APA utilization could be caused by removal of destabilizing elements, like miRNAs at the 3’-UTR of transcripts [4, 5]. It remains to be determined how those elements differ between muscles and cell cultures. Moreover, the relation between PAS and miRNA is not fully understood.

The proteasomal gene network is consistently dysregulated in OPMD muscles and in mouse and *Drosophila* models [25]. In shPab muscles reduced expression levels of some proteasomal genes was consistent with reduced proteasome activity (Fig 6). Reduced proteasome activity in shPab muscles is in agreement with previous reports in aging muscles [31]. We employed a pan-reactive activity probe and monitored proteasomal subunit-specific activity in cell cultures and in muscle cryosections. Importantly, we report consistent changes in proteasome activity using different procedures, including flow cytometer. Therefore, proteasome activity could be a robust biomarker for myogenic dysfunction at early stages of muscle atrophy and late-onset myopathies. How proteasome activity is impaired in conditions with reduced PABPN1 levels is not fully understood. Interestingly, proteasomal activity in shPab culture is not entirely abolished, as Epox treatment causes elevation in protein accumulation. This could suggest that under reduced Pabpn1 levels conditions the proteasome may undergo rearrangement of subunits, which lead to impaired activity. We show that Epox treatment resulted in a higher protein accumulation of Pabpn1, Atrogin-1 and MyHC-2b proteins in shPab muscle cells compared to control cells. This suggests that compensation in protein catabolism could be involved restoring protein levels in conditions with limited PABPN1 levels. Those mechanistic insights could open new options to understand tissue aging.

The pathophysiology of aging muscles can be recognised by multiple histological features [32]. Myofiber atrophy, central nucleation, ECM thickening and myofiber transitions were primarily detected in muscles with mild Pabpn1-DR levels (Fig 6). These histological signatures of muscle degeneration were also found in OPMD muscles [30, 33]. In addition, atrophy and an increase in MyHC-2a myofibers were also found in the A17.1 mouse mode [24]. This suggests that reduced Pabpn1-levels induce pathology similar to muscle aging and OPMD. We quantified myofiber transitions using a newly developed image quantification procedure [34]. This procedure reveals moderate changes in myofiber transitions even in mild condition of Pabpn1-DR for a short span of time. Despite the remarkable changes in MyHC isotypes expression in myofibers, mild Pabpn1-DR did not affect mRNA expression of MyHC genes. The accumulation of MyHC proteins is regulated by Murf1 and Atrogin-1 [35, 36], which we also found to be up-regulated in shPab muscles. Also in muscle cell cultures reduced PABPN1 levels cause reduced MyHC expression levels, which were elevated by proteasomal inhibitors. We therefore suggest that the changes in MyHC isotypes expression in muscle aging are mediated by Pabpn1 levels via the ubiquitin proteasome system. Indeed, Epox treatment in muscle cell culture elevated MyHC-2b and PABPN1 levels, and thus could open new options to restore muscle weakness.

In conclusion, we studied the effect of limited Pabpn1 levels in mouse muscles at 4 weeks post-Pabpn1 inhibition. These findings may represent the early effects of reduced Pabpn1 levels in aging and OPMD muscles. Mild Pabpn1-DR initially causes myofiber atrophy and transition in the expression levels of the proteins of the MyHC isotypes. Reduced Pabpn1 levels regulating APA utilization in muscles could be an early precursor for gene dysregulation affecting a subset of UPS genes, consequently leading to muscle pathology that resembles that of aging and OPMD affected muscles.

## Materials and Methods

### Ethics statement

Experiments were carried out in accordance with the ARRIVE guidelines [37], under an animal research protocol (DEC #13113), approved by the animal ethical committee, LUMC, Leiden, The Netherlands.

### Construct generation and AAV packaging

Previously described shRNA to mouse Pabpn1 [4] was cloned into the backbone of the AAV expression vector pDD2 [38] together with the eGFP gene as a reporter marker. Scramble shRNA control or shPab were expressed under the U6 promoter, and eGFP under the CMV promoter. Packaging into AAV9 serotype and virus particle production were carried out by Vector BioLabs (Malvern, Pennsylvania, USA). Viral titer is indicated as genomic copies (gc)/ml. A schematic diagram of the pDD2-AAV construct is shown in S9 Fig.

### Mouse strain, AAV injection, mice imaging and GFP quantification

Male, 7–8 week old C57BL/6J mice (Jackson laboratories, Sacramento, CA. USA) were acclimatized for one week. AAV9 particles (1.5 x 10^12^ gc in 50 μl PBS) of Pabpn1 (shPab) or scramble (Scram) shRNA were injected into left or right TA muscles (n = 5 mice), respectively. PBS-injected TA muscles were included as a non-transduced control. Injections were carried out under general anaesthesia using 2% isoflurane (Pharmachemie BV, Haarlem, The Netherlands). Mice were housed in ventilated cages with sterile bedding, water, rodent food and air in DM-III containment level.

Mice were imaged on a weekly basis for a period of 4 weeks using the Maestro^™^ in-vivo fluorescence imaging system (Xenogen product from Caliper Life Sciences, Hopkinton, Massachusetts, USA) according to manufacturer’s instructions. Prior to imaging mice were anesthetized with a continuous flow of 2% isoflurane and GFP fluorescence was acquired with an acquisition time ranging from 30 to 90 seconds depending upon the fluorescent signals from the AAV-injected TA muscles. Mean fluorescence intensity (MFI) was quantified using ImageJ, version 1.48 (National Institute of Health, USA) [39]. Four weeks post-injection, mice were sacrificed by cervical dislocation and TA muscles were collected and immersed in liquid nitrogen chilled isopentane (30–45 seconds) and stored at -80°C for *ex-vivo* analyses.

### Muscle histology

Cryosectioning (10 μm) was carried out from 4 alternating parts along the muscles, with the intervening tissue collected for RNA extraction to ensure the representation of whole muscle in all analyses (S9 Fig). Sections were made with a cryostat CM3050S (Leica Microsystems) and pasted on super frost plus glass slides (Menzel-Glaser; Thermo scientific), and were stained with haematoxylin and eosin (H&E) [40]. GFP distribution in myofibers was carried out in unfixed cryosections.

Immunofluorescence was carried out in unfixed cryosections incubated with a primary antibody to laminin (1:1000; ab11575, Abcam) and hybridoma 6H1 detecting MyHC-2x (1:5; Developmental Studies Hybridoma Bank (DSHB)). Subsequently, appropriate secondary anti-rabbit-Alexa-647-conjugated and anti-mouse-Alexa-564-conjugated (Molecular Probes, Invitrogen) were applied. After extensive washing muscle sections were incubated with a mixture of monoclonal antibodies to MyHC-1, MyHC -2a and MyHC -2b proteins (BA-D5, SC-71 and BF-F3, respectively (DSHB)), which were conjugated with Alexa-350, Alexa-594 and Alexa-488, respectively. Conjugation was carried out as described in [41]. Immunofluorescence with Goat-anti-collagen 1 (1:200, # 1310–01, Southern Biotech), and staining with Nile Red were carried out in unfixed cryosections as described in [42].

Epoxomicin (0.5μM) pre-incubation was carried out for 1 hour, and LWA300 incubation (130 nM) in PBS was carried out for 40 minutes in cryosections. Subsequently the probe was washed three times with PBS, EDTA, and methanol:PBS (1:1). Finally, sections were dehydrated with ethanol and mounted in Citifluor (Le, UK) containing DAPI. EDTA and methanol washes removed non-specific LWA300 staining and GFP signal (S3 Fig). LWA300 signal was quantified from the GFP positive fibers in sequential sections. Slides were mounted with Aqua Polymount (Polyscience), and where indicated DAPI was included.

### Cell culture

Immortalized mouse myoblasts, C2C12, were propagated as described in [4]. Pabpn1-DR cultures were generated with shRNA expressed by a lentivirus expression system as described in [4]. Epoxomicin treatment was carried out at 0.5–0.05 μM in myoblasts for 1 hour, as indicated per experiment. Mock treatment was carried out with 0.001% DMSO. The human myoblasts, 7304.1, stably express empty vector (H1 control) or shRNA to PABPN1 (shPAB), were previously described in [27]. Myoblasts were seeded in 96 well plates. Fusion was carried out in 2% horse-serum for 3 days, and Epox treatment (50nM) was carried out in 3 days fused culture for 1 hour. Cycloheximide treatment (50μg/ml) was carried out for 0, 1, 3, 5 hours.

### RNA analyses

Total RNA was isolated from mouse muscles using miRNeasy Mini Kit (QIAGEN), whilst RNA from C2C12 cell cultures RNA was isolated using acid phenol extraction (Qiazol, QIAGEN). cDNA synthesis was carried out using the RevertAid First Strand cDNA Synthesis Kit (ThermoScientific). 3ng cDNA was used as template per PCR reaction with specific primers (S2 Table). Quantitative PCR was carried out using iQ-SYBR Green (Bio-Rad Laboratories) and LightCycler 480 System (Roche Diagnostics). Fold change was calculated after normalisation to the *Hprt* housekeeping gene and to the non-transduced cells or to scramble injected muscles where appropriate. Utilization of distal PAS was determined with a primer set that amplifies long transcripts that are generated from distal PAS. The proximal primer set amplifies all transcripts with short or long 3’-UTR. A change in PAS utilization was calculated by the ratio distal/proximal. Fold change was determined with a primer set covering last exon and the 3’-UTR. Primers are listed in S2 Table.

### Western blot analyses and immunofluorescence

Proteins were extracted with RIPA buffer followed by sonication; 30 μg aliquots were loaded on SDS-PAGE and western blot was carried out on PVDF membrane and protein levels were determined using the immunoblot procedure.

Fused cultures of 7304 human myoblast were fixed with 2% formaldehyde in PBS and permeabilized with 1% triton prior to antibody incubation. Myoblasts were incubated with a mixture of conjugated antibodies to MyHC-2a (Alexa-594), MyHC-2x (Alexa-564), MyHC-2b (488) as previously described [34] and DAPI was added to counter stain nuclei.

Antibodies used in this study are: anti-PABPN1 (1:2000; anti-rabbit, LS-B8482, LS Bio, WA. USA), PSMD14 (1:1000; anti-rabbit, #7662, Cell Signalling, MA. USA), proteasome β-subunit 2 (PSB2) (1:1000, anti-mouse, sc-58410, Santa Cruz, CA, USA), proteasome β-subunit 5 (PSB5) (1: 1000; anti-rabbit, #09–278, Millipore, MA, USA), PA28α (1:1000; anti-rabbit, #2408, Cell Signalling, MA. USA), Atrogin1 (1:1000, anti-rabbit, # AP2041, ECM Bioscience), Murf1 (1: 1500, anti-rabbit, # MP3401, ECM Bioscience) and anti-mouse tubulin (1:2000, clone DM1A Sigma-Aldrich). Glyceraldehyde 3-phosphate dehydrogenase Gapdh 1:10,000 dilution; anti-mouse G8795, Sigma-Aldrich), Actin (1:1000) or coomassie blue stained gels were used as loading controls. Primary antibodies were detected with a secondary IRDye 800CW or IRDye 680RD conjugated (Licor, NE. USA). Fluorescent signals were detected using the Odyssey CLx Infrared imaging system (Licor). Quantification of protein accumulation was performed with ImageJ. Values were corrected for background and normalized to loading controls.

### Measurement of proteasome activity using pan-reactive probes

The activity of the catalytic β-subunits (β1, β2 & β5) in C2C12 cell cultures was measured using the pan-reactive activity based probe (ABP) LWA300 (125 nM), an Epox-derived probe conjugated to the commercial fluorophore, Bodipy-FL [28]. The specificity of the LWA300 probe was assessed following pre-incubation with Epox (Sigma-Aldrich, MO, USA) (0.5μM) in proliferation medium. Cell cultures were lysed and proteins were separated on denaturing SDS-PAGE gels. LWA300-bound proteins were visualised using BioRad ChemiDoc imaging systems (BioRad, CA, USA). MFI of protein bands were normalised to proteins in coomassie blue stained gels.

For cell cytometry, myoblast cultures were incubated with 62.5nM LWA300 for 1 hour. Cells were trypsinized and washed with 50mM EDTA (1x) and stained with Hoechst prior to analysis. Data were collected using the BD-LSR II flow cytometer and BD FACSDiva software. Events were gated on forward and side scatters (FSC and SSC; total 15,000 events) and subsequently only Hoechst positive events were analysed for LWA300 MFI (average gated live population of 12,869 ±156). Data analysis was carried out using FlowJo software suite (FlowJo, OR, USA). LWA300 fluorescence in cells was imaged from cells seeded on glass. LWA300 treatment was carried out as described for the flow cytometry here above, but following EDTA wash cells were also washed with Methanol:PBS (1:1) and mounted with Citifluor containing DAPI.

### Imaging and image quantification

Imaging was carried out with DM5500 (Leica Microsystems) for visualization of cell cultures or with TCS-SP5 confocal (Leica Microsystems) for visualization of MyHC isotypes in muscle sections (S6 Fig). Fluorescent images were captured with the LAS AF software versions: 2.3.6 or 2.5.1.6757 for the DM5500 or the TCS-SP5, respectively and H&E images were captured using a LAS software version: 4.5.0 for the DM-LB light microscope.

Imaging of GFP was carried out in untreated sections that were directly mounted with Polymount containing DAPI. Both GFP and LWA300 were imaged with a FITC filter. The MFI of all four MyHC isotypes in myofibers was quantified as previously described [34] using an in-house developed procedure (S10 Fig). In brief, the laminin image was thresholded and inversed resulting in segmented myofibers. In objects where the contour lines had missing pixels, a continuous line was extrapolated as ultimate erosion by shrinking the areas into a closed object and then expanding it to its original size. Subsequently, from each segmented object the MFI for all four MyHC isotopes was measured together with the myofiber cross-sectional area. The MFI values were corrected for background, normalized for the fluorophores and normalized for the sum of the MFI for each myofiber. Distribution plots were generated after sorting MyHC MFI within myofibers: MyHC-2b from large to small and on MyHC-2x from small to large. Cumulative distribution plots were generated for each MyHC separately. The MFI values were then used for subsequent analyses of myofiber composition, and detailed in the statistical section. LWA300 MFI was measured in ImageJ. For quantification of nuclear LWA300 MFI, the nucleus was segmented from the DAPI image. Quantification of collagen1 was carried out in ImageJ: collagen1 signal was thresholded using a fixed value which was applied for all images, and the % of collagen1 positive area was calculated from the total field area (pixel/μm^2^). An example of a thresholded image is shown in S11 Fig.

Imaging of fused myoblasts (myotubes) was carried out with the ArrayScan VTI HCA (Thermofisher, Waltham, Massachusetts, USA) using a 10x objective. Image segmentation and quantification was carried out using the ArrayScan VTI HCA software. Nuclei were first segmented on DAPI and MyHC expression was then segmented on the FITC, TRITC, TexasRed channels, respectively for MyHC-2b, -2x and -2a. MFI in the cytoplasmic compartment was extracted in regions excluding the segmented nuclei. The expression of MyHC-2a and MyHC-2x was below detection levels, and was excluded from image quantification. Mean fluorescent intensities (MFIs) were determined from an average of 3760 ± 1870 myoblasts per condition.

### Statistical analysis

Paired t-test analyses were carried out to compare shPab and Scram. Unpaired t-tests were carried out to assess statistical significance in tissue culture experiments. Cumulative distribution plots were assessed with the Kruskal-Wallis test. Co-expression correlations were assessed with Spearman’s rank correlation coefficients, with subsequent paired t-tests to assess differences in co-expression between paired Scram and shPab conditions. Statistical analyses were performed with IBM SPSS Statistics 20 or in Graphpad Prism 6. P-values <0.05 were considered statistical significant. Charts were generated in Graphpad Prism 6.

## Supporting Information

S1 Fig Paired analysis of fold change and PAS utilization in five AAV9-injected mice. (PDF)

S2 Fig MFI LWA300 in living cells. (PDF)

S3 Fig Muscle histology with LWA300. (PDF)

S4 Fig Histological and molecular analyses of shPAB and Scram TA. (PDF)

S5 Fig Protein accumulation in cycloheximide-treated muscle cell cultures. (PDF)

S6 Fig Immunohistochemistry with antibodies to four MyHC isotypes. (PDF)

S7 Fig MFI distribution plots for four MyHC isotypes. (PDF)

S8 Fig mRNA expression analysis of MyHC genes in TA muscles. (PDF)

S9 Fig A schematic diagram of viral construct and muscle. (PDF)

S10 Fig Main steps in automatic image quantification using in-house ‘stack’ program. (PDF)

S11 Fig Example of collagen-1 staining and thresholded images. (PDF)

S1 Table Spearman's correlations between myosin heavy chain isotypes in scramble and shRNA injected mouse muscles. (PDF)

S2 Table Primer list and their sequences. (PDF)
