# Supplementary material for: PABPN1-Dependent mRNA Processing Induces Muscle Wasting
Source: PLoS Genet. 2016 May 6;12(5):e1006031. doi: 10.1371/journal.pgen.1006031 (PMC4859507; doi:10.1371/journal.pgen.1006031)
Supplement: S1 Fig — (PDF) [file pgen.1006031.s001.pdf]

S1 Fig. Paired analysis of fold change and PAS utilization in five AAV9-injected mice.

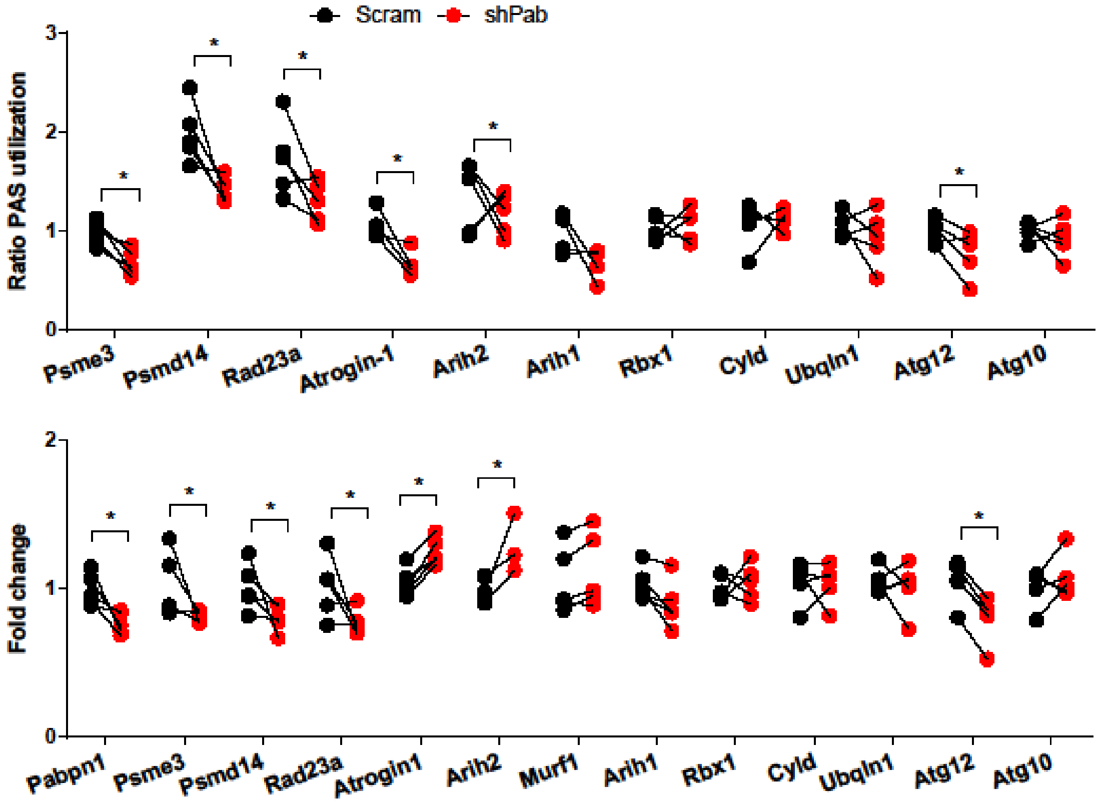

A. PAS utilization is measured by the ratio of distal to total levels per gene. **B**. mRNA fold changes are calculated by normalizing to Hprt levels and to the Scram muscles. Statistical significance is assessed by a paired Student's t-test (*: $p<0.05$).
