# Supplementary material for: PABPN1-Dependent mRNA Processing Induces Muscle Wasting
Source: PLoS Genet. 2016 May 6;12(5):e1006031. doi: 10.1371/journal.pgen.1006031 (PMC4859507; doi:10.1371/journal.pgen.1006031)
Supplement: S2 Fig — (PDF) [file pgen.1006031.s002.pdf]

**S2 Fig. MFI LWA300 in living cells.**

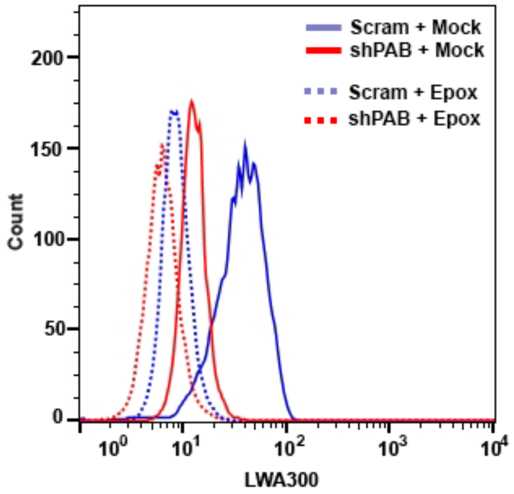


Histograms of LWA300 MFI from a representiative experiment are depicted. Dotted lines show MFI Epox trated cultures and Mock treated cultures with solid lines. Control cultures are in blue and shPab culture in red.
