# Supplementary material for: PABPN1-Dependent mRNA Processing Induces Muscle Wasting
Source: PLoS Genet. 2016 May 6;12(5):e1006031. doi: 10.1371/journal.pgen.1006031 (PMC4859507; doi:10.1371/journal.pgen.1006031)
Supplement: S3 Fig — (PDF) [file pgen.1006031.s003.pdf]

S3 Fig. Muscle histology with LWA300.

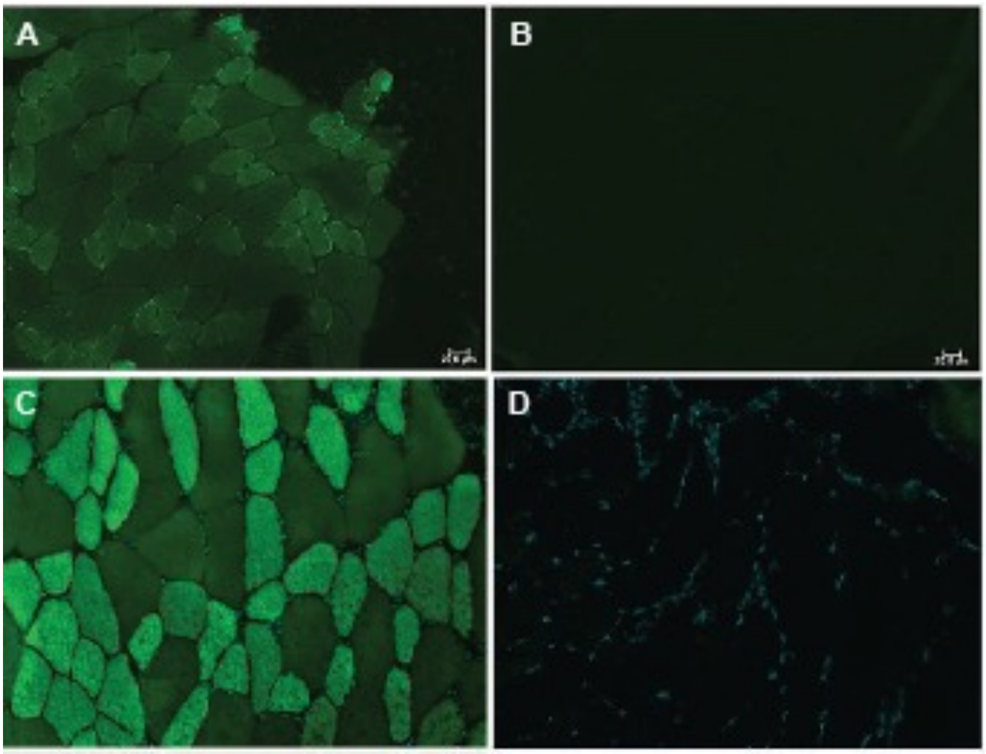

A-B. LWA300 signal in tibialis anterior muscles. Images show muscle cryosections after incubation with LWA300 (A) or LWA300 after Epox pre-incubation (B). **C**. EGFP signal in transduced muscle section prior to washings. **D.** EGFP signal after PBS, EDTA and methanol:PBS (1:1) washings. DAPI counterstains the nuclei.
