# Supplementary material for: PABPN1-Dependent mRNA Processing Induces Muscle Wasting
Source: PLoS Genet. 2016 May 6;12(5):e1006031. doi: 10.1371/journal.pgen.1006031 (PMC4859507; doi:10.1371/journal.pgen.1006031)
Supplement: S4 Fig — (PDF) [file pgen.1006031.s004.pdf]

S4 Fig. Histological and molecular analyses of shPAB and Scram TA muscles.

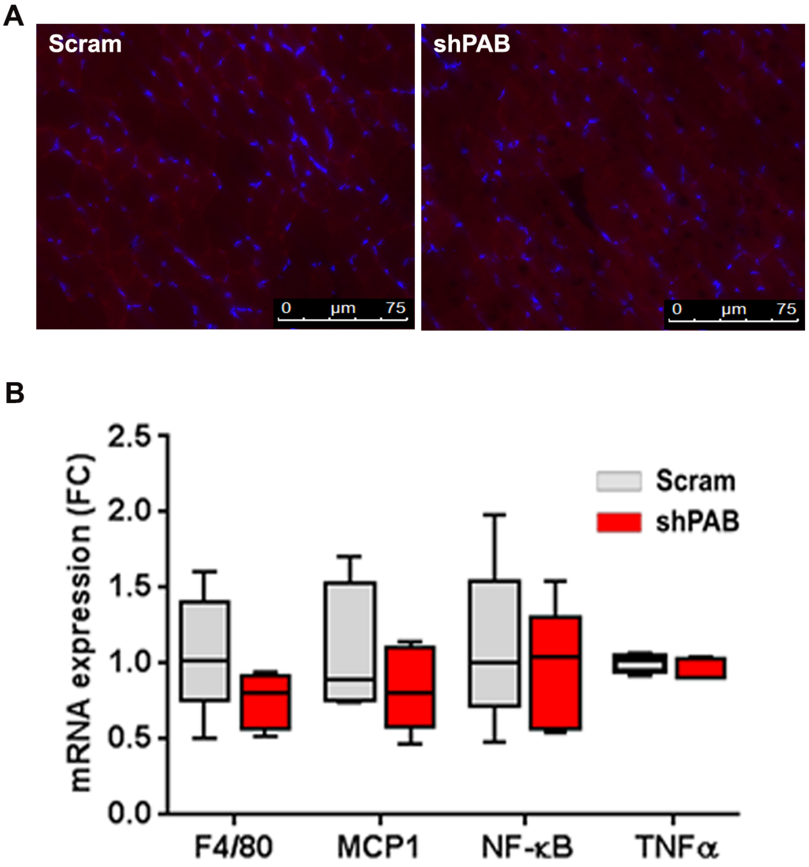

A. Representative images of Nile-Red staining for fatty droplets. Nuclei are counterstained with DAPI. **B.** mRNA expression of four inflammatory genes. CT values are normalized to *Hprt* house-keeping gene and data is presented in fold-change after normalization to PBS injected TA muscles. Measurements are from five animals.
