# Supplementary material for: PABPN1-Dependent mRNA Processing Induces Muscle Wasting
Source: PLoS Genet. 2016 May 6;12(5):e1006031. doi: 10.1371/journal.pgen.1006031 (PMC4859507; doi:10.1371/journal.pgen.1006031)
Supplement: S5 Fig — (PDF) [file pgen.1006031.s005.pdf]

S5 Fig. Protein accumulation in cycloheximide-treated muscle cell cultures.

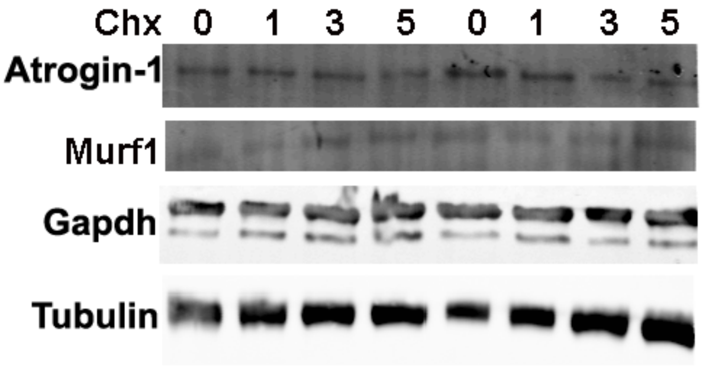

C2C12 Scram or shPab cell cultures were treated with cycloheximide (Chx) for 0, 1, 3, 5 hours. Shown are blots from a representative experiment. Equal laoding was assed by Gapdh and Tubulin.
