# Supplementary material for: PABPN1-Dependent mRNA Processing Induces Muscle Wasting
Source: PLoS Genet. 2016 May 6;12(5):e1006031. doi: 10.1371/journal.pgen.1006031 (PMC4859507; doi:10.1371/journal.pgen.1006031)
Supplement: S6 Fig — (PDF) [file pgen.1006031.s006.pdf]

S6 Fig. Immunohistochemistry with antibodies to four MyHC isotypes. and laminin.

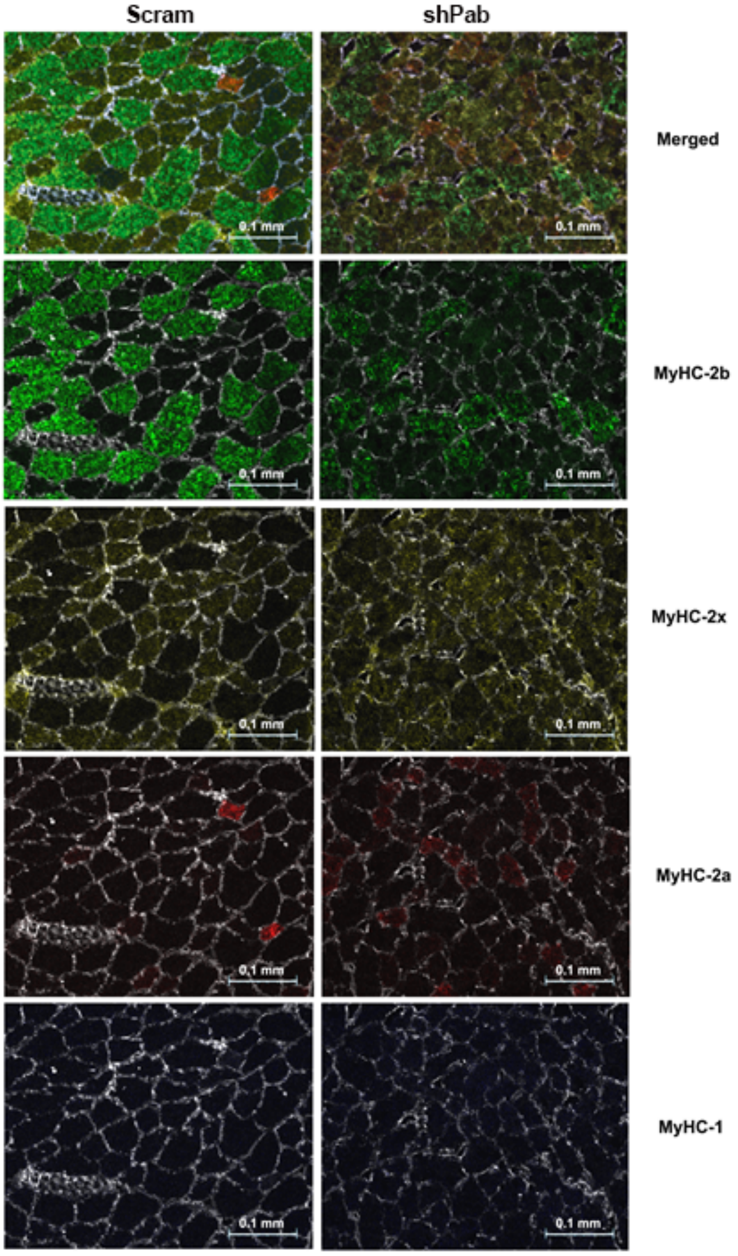

Representative images of cross-sections stained with an antibody mix to four MyHC isotypes (-2b in green, -2x in yellow, -2a in red and type-1 in blue) in Scram or shPAB TA muscles. Sections were co-stained with anti-laminin (white).
