# Supplementary material for: PABPN1-Dependent mRNA Processing Induces Muscle Wasting
Source: PLoS Genet. 2016 May 6;12(5):e1006031. doi: 10.1371/journal.pgen.1006031 (PMC4859507; doi:10.1371/journal.pgen.1006031)
Supplement: S7 Fig — (PDF) [file pgen.1006031.s007.pdf]

S7 Fig. MFI distribution plots for four MyHC isotypes.

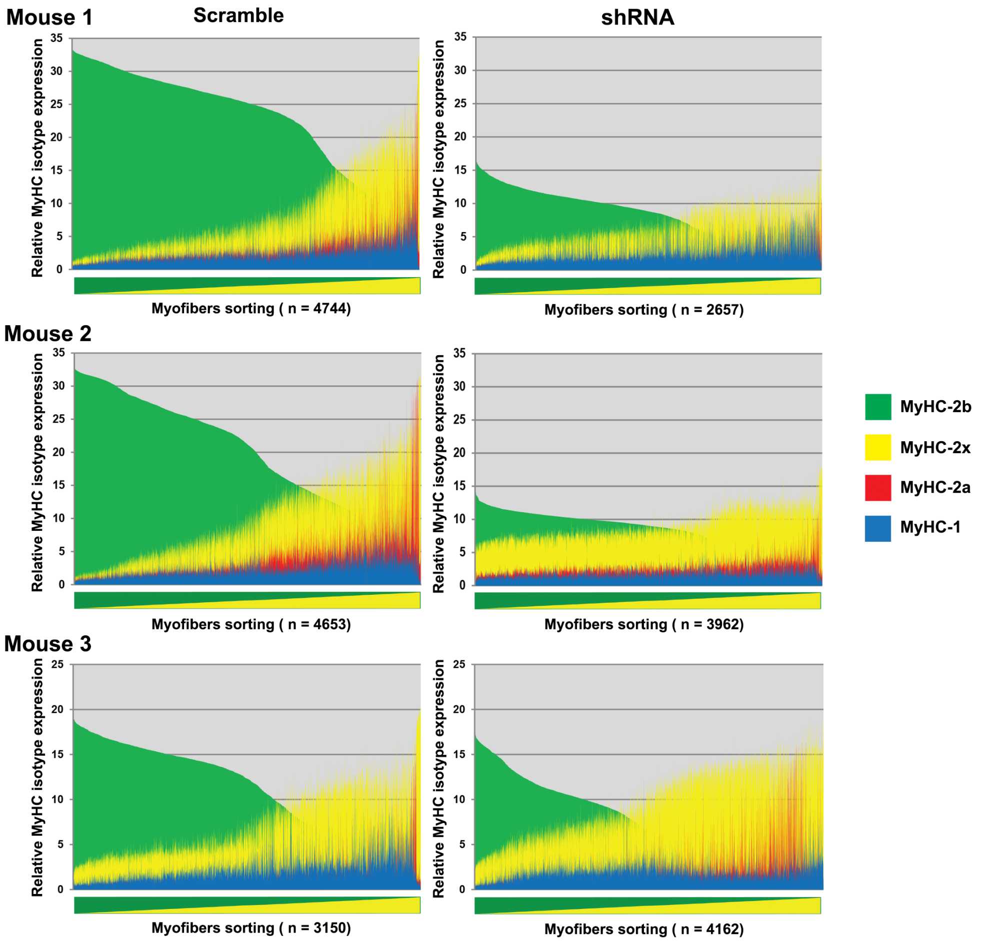

Plots are from three mice with a high Pabpn1 down-regulation (20-50%). Y-axis show MFI, and X-axis shows myofibers sorted by MyHC-2b high to low and MyHC-2x low to high.
