# Supplementary figures and images for: PABPN1-Dependent mRNA Processing Induces Muscle Wasting
Source: PLoS Genet. 2016 May 6;12(5):e1006031. doi: 10.1371/journal.pgen.1006031 (PMC4859507; doi:10.1371/journal.pgen.1006031)

**S8 Fig. mRNA expression analysis of MyHC genes in TA muscles.**

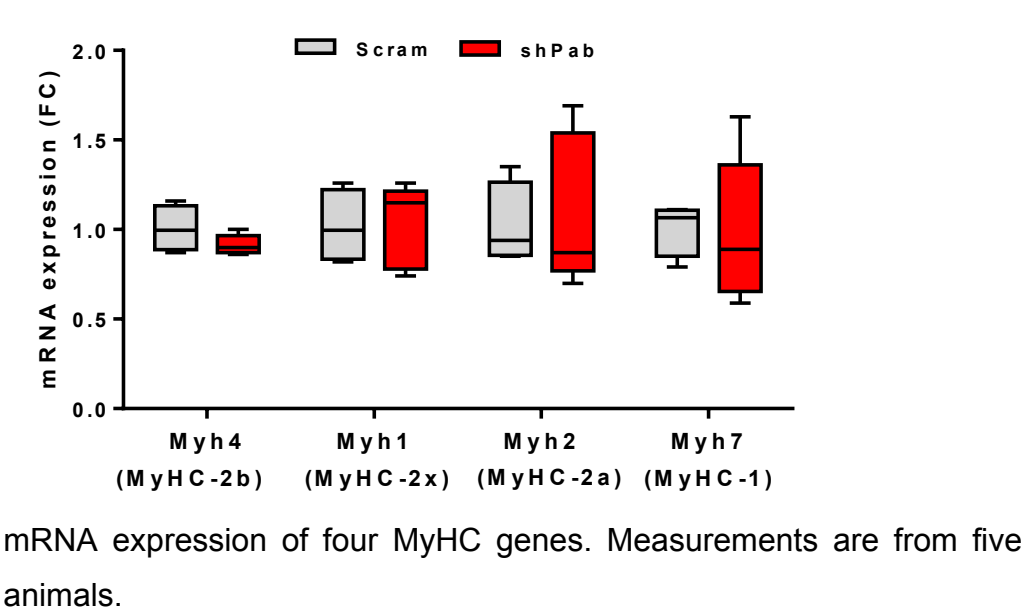

Supplement: S8 Fig — (PDF) [file pgen.1006031.s008.pdf]
