# Supplementary material for: PABPN1-Dependent mRNA Processing Induces Muscle Wasting
Source: PLoS Genet. 2016 May 6;12(5):e1006031. doi: 10.1371/journal.pgen.1006031 (PMC4859507; doi:10.1371/journal.pgen.1006031)
Supplement: S9 Fig — (PDF) [file pgen.1006031.s009.pdf]

S9 Fig. A schematic diagram of viral construct and muscle.

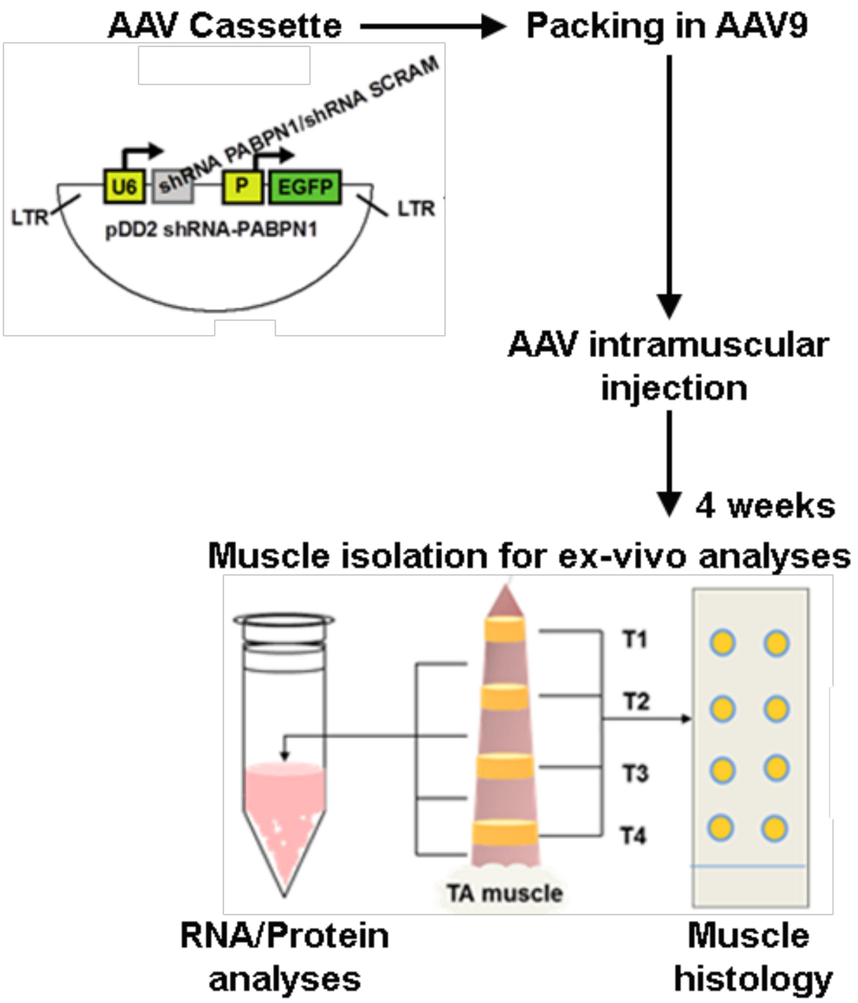

analyses.

The shRNA to PABPN1 was cloned into the backbone of pDD-2 vector under the U6 promoter. Scramble shRNA was used as a control. EGFP, a reporter for AAV transduction, was cloned under the CMV promoter ("P" in the vector map scheme). AAV9 particles were generated and injected into mouse TA muscles. Mice were sacrificed and muscles were harvested and stored at -80˚C prior to *ex-vivo* analyses. Transverse cryosections were made from 4 regions covering the whole muscle and from the intervening regions RNA or proteins were extracted.
