# Supplementary material for: PABPN1-Dependent mRNA Processing Induces Muscle Wasting
Source: PLoS Genet. 2016 May 6;12(5):e1006031. doi: 10.1371/journal.pgen.1006031 (PMC4859507; doi:10.1371/journal.pgen.1006031)
Supplement: S10 Fig — (PDF) [file pgen.1006031.s010.pdf]

S10 Fig. Main steps in automatic image quantification using in-house 'stack' program.

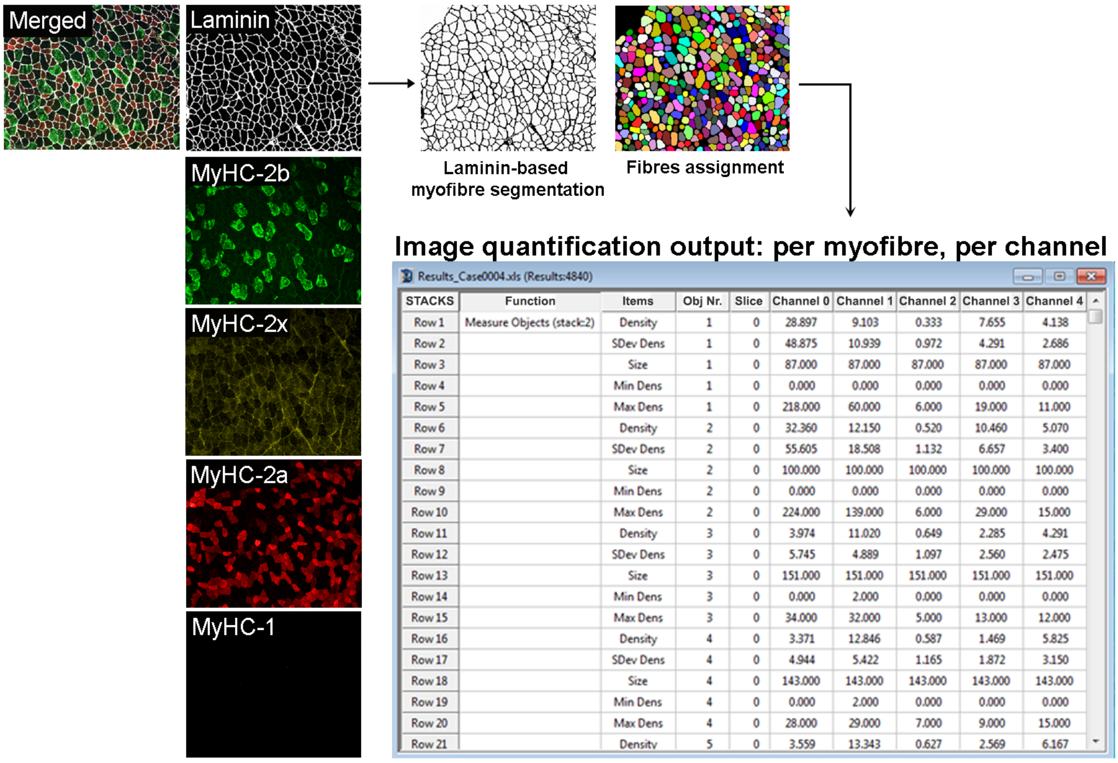

STACKS	Function	Items	Obj Nr.	Slice	Channel 0	Channel 1	Channel 2	Channel 3	Channel 4
Row 1	Measure Objects (stack:2)	Density	1	0	28.897	9.103	0.333	7.655	4.138
Row 2		SDev Dens	1	0	48.875	10.939	0.972	4.291	2.686
Row 3		Size	1	0	87.000	87.000	87.000	87.000	87.000
Row 4		Min Dens	1	0	0.000	0.000	0.000	0.000	0.000
Row 5		Max Dens	1	0	218.000	60.000	6.000	19.000	11.000
Row 6		Density	2	0	32.360	12.150	0.520	10.460	5.070
Row 7		SDev Dens	2	0	55.605	18.508	1.132	6.657	3.400
Row 8		Size	2	0	100.000	100.000	100.000	100.000	100.000
Row 9		Min Dens	2	0	0.000	0.000	0.000	0.000	0.000
Row 10		Max Dens	2	0	224.000	139.000	6.000	29.000	15.000
Row 11		Density	3	0	3.974	11.020	0.649	2.285	4.291
Row 12		SDev Dens	3	0	5.745	4.889	1.097	2.560	2.475
Row 13		Size	3	0	151.000	151.000	151.000	151.000	151.000
Row 14		Min Dens	3	0	0.000	2.000	0.000	0.000	0.000
Row 15		Max Dens	3	0	34.000	32.000	5.000	13.000	12.000
Row 16		Density	4	0	3.371	12.846	0.587	1.469	5.825
Row 17		SDev Dens	4	0	4.944	5.422	1.165	1.872	3.150
Row 18		Size	4	0	143.000	143.000	143.000	143.000	143.000
Row 19		Min Dens	4	0	0.000	2.000	0.000	0.000	0.000
Row 20		Max Dens	4	0	28.000	29.000	7.000	9.000	15.000
Row 21		Density	5	0	3.559	13.343	0.627	2.569	6.167

Muscle sections were immunostained for four MyHC isotypes and laminin. Myofibers were segmented from the laminin images. The mean fluorescence intensity (MFI) and cross sectional area from all five channels (Channel 0 = laminin, Channel1 = MyHC-2b, Channel 2 = MyHC-2x, Channel 3 = MyHC-2a, and Channel 4 = MyHC-1) was documented per myofiber.
