# Supplementary material for: PABPN1-Dependent mRNA Processing Induces Muscle Wasting
Source: PLoS Genet. 2016 May 6;12(5):e1006031. doi: 10.1371/journal.pgen.1006031 (PMC4859507; doi:10.1371/journal.pgen.1006031)
Supplement: S11 Fig — (PDF) [file pgen.1006031.s011.pdf]

S11 Fig. Example of collagen-1 staining and thresholded images.

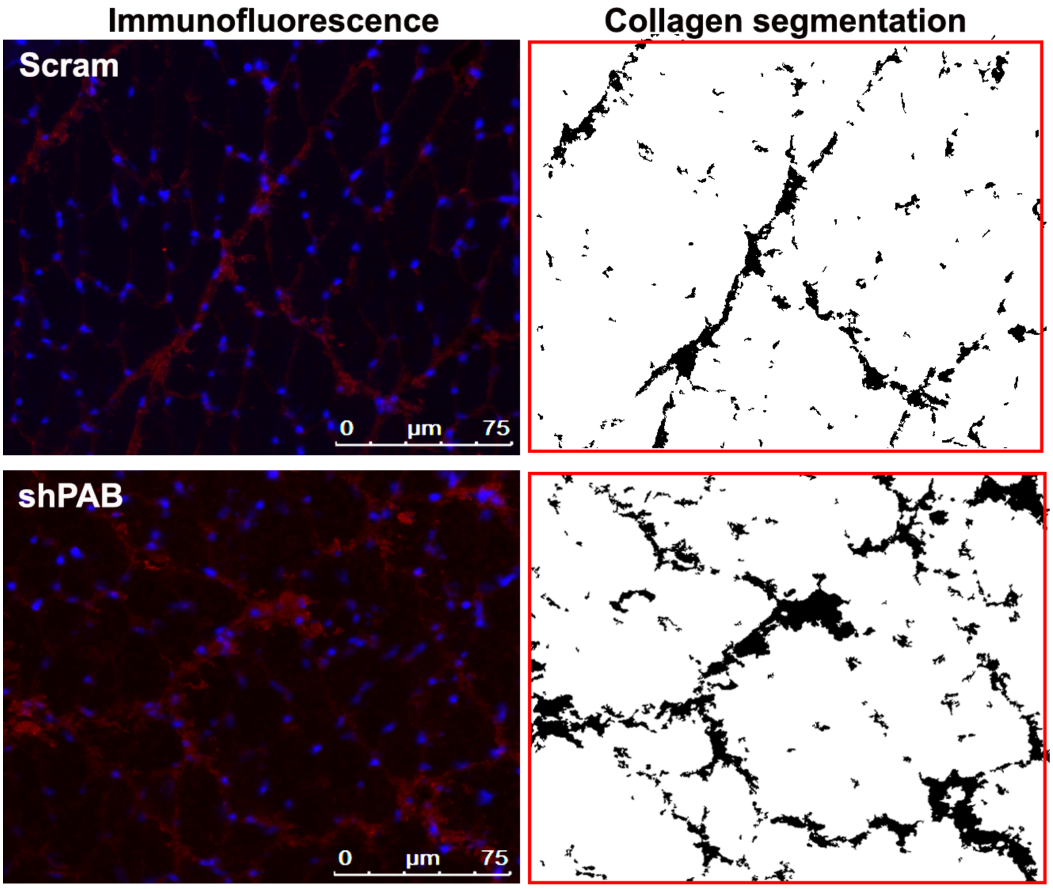

Left panel (Immunofluorescence) shows representative images of Collagen1 is shown in red, and nuclei in blue in Scram and shPab muscles. Right panel (Collagen segmented) shows the corresponding segmented image. Segmentation was carried out on collagen1 staining.
