# Supplementary material for: PABPN1-Dependent mRNA Processing Induces Muscle Wasting
Source: PLoS Genet. 2016 May 6;12(5):e1006031. doi: 10.1371/journal.pgen.1006031 (PMC4859507; doi:10.1371/journal.pgen.1006031)
Supplement: S1 Table — (PDF) [file pgen.1006031.s012.pdf]

S1 Table. Spearman's correlations between myosin heavy chain isotypes in scramble and shRNA injected mouse muscles.

	Scramble injected muscles			shRNA injected muscles			
	Mouse 1 (N=4744)	Mouse 2 (N=4653	Mouse 3 (N=3200	Mouse 1 (N=2657	Mouse 2 (N=3962	Mouse 3 (N=4162	p-value for change[$]
MyHC-2b - MyHC-2x	-0.92	-0.93	-0.87	-0.83	-0.70	-0.85	0.21
**MyHC-2b - MyHC-2a**	-0.89	-0.89	-0.80	-0.64	-0.69	-0.64	**0.02**
MyHC-2b - MyHC-1	-0.64	-0.66	-0.63	-0.30	-0.55	-0.20	0.09
**MyHC-2x - MyHC-2a**	0.73	0.73	0.57	0.33	0.22	0.33	**0.04**
**MyHC-2x - MyHC-1**	0.50	0.44	0.32	-0.05	-0.03	0.04	**0.03**
MyHC-2a - MyHC-1	0.65	0.75	0.71	0.24	0.66	0.35	0.10

Spearman's correlation indices on ln-transformed data are provided. Non-significant correlations are depicted in grey.

N = indicates the number of myofibers quantified per mouse

[$] = A paired Student’s T test was used to compare Spearman correlation indices between a pair of MyHC isotypes in Scramble and shRNA injected TA muscles Significant changes in Spearman’s correlations are depicted in bold.
