# Supplementary material for: PABPN1-Dependent mRNA Processing Induces Muscle Wasting
Source: PLoS Genet. 2016 May 6;12(5):e1006031. doi: 10.1371/journal.pgen.1006031 (PMC4859507; doi:10.1371/journal.pgen.1006031)
Supplement: S2 Table — (PDF) [file pgen.1006031.s013.pdf]

S2 Table. Primer list and their sequences

Gene	Ensemble ID	Forward 5'-3'	Reverse 5'-3'
**Hprt1**	ENSMUSG00000025630	CGTCGTGATTAGCGATGATG	TTTTCCAAATCCTCGGCATA
**Pabpn1**	ENSMUSG00000022198	GCCTCACTTTTGAGGTGCTC	AGGGGAATACCATGATGTCG
**Murf1**	ENSMUSG00000028834	TCGTTCGAGCCTTACCTGTT	AAGTGAAGCTGGCAGGTTCAA
**Atrogin-1 (Fbxo32) Proximal**	ENSMUSG00000022358	TGTGCTGGTGGGCAACATTA	GGTGATCGTGAGGCCTTTGA
**Atrogin-1 (Fbxo32) Distal**	ENSMUSG00000022358	CGTGTTAACTATGCACAGGGC	GCACATAATCTGTCCAACCTTCC
**Psme3 Proximal**	ENSMUSG00000078652	CCAGTTTTCCCATCACGATA	GAGCAGCAATGCAGAGACAC
**Psme3 Distal**	ENSMUSG00000078652	GGCAGGCTAATTGCAGAGAC	GCGAAGGTCAAACCCATAGA
**Psmd14 Proximal**	ENSMUSG00000026914	GAATGGGATCCACAACCACT	ATCACAGTCACCCTGGCTTT
**Psmd14 Distal**	ENSMUSG00000026914	TGATGTATTCCTGAACAACTTTGG	TGGTGATGTGTGAGACACCTG
**Rad23 Proximal**	ENSMUSG00000003813	ATGAGAATCTGGCTGCCAAC	ACCCCCACCAACCTACTACC
**Rad23 Distal**	ENSMUSG00000003813	CCTCCCCTCCGTAGAGATGT	TTCCACCCACCTTCTCTTTG
**Arih2 Proximal**	ENSMUSG00000064145	TTCTGCAACTTTGCTGGATG	CTGCTCCACATCACTGGCTA
**Arih2 Distal**	ENSMUSG00000064145	TGGAGGCTGAGATCGAAAAC	GCTGCTCTGCTATGTGCATC
**Arih1 Proximal**	ENSMUSG00000025234	GTCCAAACCAGCCATTTTGT	CTATCCCCTTCTCCCTCCAA
**Arih1 Distal**	ENSMUSG00000025234	GGAAAACGGCTCCTTCATAA	GGTTGGTTAATTCAGCTTGAAAA
**Rbx1 Proximal**	ENSMUSG00000022400	CACTGCATCTCTCGATGGCT	GGGTACGCCTTGCGGGAAAG
**Rbx1 Distal**	ENSMUSG00000022400	GAACGAGTGACTGGCATCAA	TGCCAGCCAAGATTACACAG
**Ubqln1 Proximal**	ENSMUSG00000005312	ACATCAATGCAGCAATCGAA	ACATCAATGCAGCAATCGAA
**Ubqln1 Distal**	ENSMUSG00000005312	GTTGAATGTCGTGCTGTTCG	GGCTCTTTTTCACCCAAATG
**Atg10 Proximal**	ENSMUSG00000021619	AAGCAACATCACAATCGGAGT	CTATCCCCTTCTCCCTCCAA
**Atg10 Distal**	ENSMUSG00000021619	CGCGTGTCTGTGGTGTGTAT	AACAGAGCAGTCGTGGGTCT
**Atg12 Proximal**	ENSMUSG00000032905	CCCAGACCAAGAAGTTGGAA	CAGCACCGAAATGTCTCTGA
**Atg12 Distal**	ENSMUSG00000032905	TAGAGGAGCCTCAGCCATGT	ACGGCCCAGTTTTCAAGATA
**Cyld Proximal**	ENSMUSG00000036712	GCCTGGCTTTTCTTTGACAG	AAGAGTGCAGGTCCTCCAGA
**Cyld Distal**	ENSMUSG00000036712	TCAAAGGTGTCAGAGCATGG	TCCCCTAACATCTCCATCCA
**Myh1**	ENSMUSG00000056328	ACCTGGCCAAATTCCGCAAG	TGGTCACTTTCCTGCACTTG
**Myh2**	ENSMUSG00000033196	TGAACTGGAGGGTGAGGTAG	TCTGGTAAGTAAGCTCCTTC
**Myh4**	ENSMUSG00000057003	GAGTGAAGGAACTCACCTAC	TTTCACTTTAGTCTGTAGTTTG
**Myh7**	ENSMUSG00000053093	AAGGGCCTGAATGAGGAGTA	GCTTCCACCTAAAGGGCTGT
**Tnnt1**	ENSMUSG00000064179	GCTGGGAAGGGTCGAGTTG	GGGCACTTTATTTTGAGTTAC
**F4/80**	ENSMUSG00000004730	CTTTGGCTATGGGCTTCCAGTC	GCAAGGAGGACAGAGTTTATCGTG
**MCP-1**	ENSMUSG00000035385	CACTCACCTGCTGCTACTCA	GCTTGGTGACAAAAACTACAGC
**TNFα**	ENSMUSG00000024401	GATCGGTCCCCAAAGGGATG	CACTTGGTGGTTTGCTACGAC
**NF-κB**	ENSMUSG00000028163	CAGCTTTACAGAGTCTCTTAC	CTTTGGATTCGCTTTGCCTTC
**EGFP**	AAV-CMV-GFP vector	GGTCGGGGTAGCCGCTGAAG	AGCTGGACGGCGACGTAAAC
